# Experimental estimation of copper-site geometry reproducibility in biologically relevant redox and saccharide-bound states of a model lytic polysaccharide monooxygenase

**DOI:** 10.1107/S2059798326005966

**Published:** 2026-07-17

**Authors:** Zhiyu Huang, Qiuyi Wei, Jie Nan, Morten H. H. Nørholm, Zimeng Liu, Cristina Hernández-Rollán, Katja S. Johansen, Leila Lo Leggio

**Affiliations:** a Department of Chemistry University of Copenhagen Universitetsparken 5 2100Copenhagen Denmark; b MAX IV Laboratory Fotongatan 2 224 84Lund Sweden; c The Novo Nordisk Foundation Center for Biosustainability Technical University of Denmark Kemitorvet Building 22 2800Kongens Lyngby Denmark; d Department of Biochemistry University of Cambridge Hopkins Building, Tennis Court Road CambridgeCB2 1QW United Kingdom; Institut de Biologie Structurale, France

**Keywords:** lytic polysaccharide monooxygenases, copper active-site geometry, X-ray crystallography, ANOVA, Tukey–Kramer *post hoc* test and *t*-test

## Abstract

Lytic polysaccharide monooxygenases (LPMOs) are copper-dependent enzymes classified into the auxiliary activity (AA) families of the CAZy database. They oxidatively cleave glycosidic bonds in recalcitrant polysaccharides, playing a key role in biomass degradation and contributing to the virulence of some pathogens. The redox state of the active-site copper and its coordination geometry are central to LPMO catalysis, yet the subtle structural consequences of redox and substrate-binding transitions remain insufficiently resolved. In previous work, a comparative X-ray crystallographic analysis of a model AA9 LPMO (LsAA9A) was conducted under four distinct conditions at 100 K: Cu(II), Cu(I) and the corresponding saccharide-bound states, with the Cu(I) state generated by X-ray photoreduction. In this study, LsAA9A crystals were chemically reduced with or without saccharide substrate prior to low-dose X-ray data collection to minimize radiation damage. Copper-coordination distances and angles were determined precisely through triplicate structure determinations (each from an independent crystal) for each condition, revealing small but reproducible geometry differences across key states in the LPMO catalytic pathway. In order to identify the most significant differences, statistical evaluation using one-way analysis of variance (ANOVA), followed by Tukey–Kramer *post hoc* tests and pairwise *t*-tests, was carried out. Within the assumptions made, statistically significant differences in the coordinated Cu–His1 N^δ1^ and Cu–Tyr O^η^ distances, and in the coordination angles θ_2_, θ_3_ and θ_T_, are observed across the four states and are discussed in terms of the mechanism and in relation to our previous study. To complement cryogenic data, multi-crystal data sets at increasing X-ray dose were collected at room temperature to track photoreduction of the copper site, with the specific aim of detecting the transition of Cu(II) to fully reduced Cu(I). This could not finally be achieved due to the onset of global radiation damage; however, a subset of the reduction-linked geometric changes were detectable, indicating that a partial transition may have occurred.

## Abstract 2

Small but measurable changes in the coordination geometry of a lytic polysaccharide monooxygenase copper active site are revealed by X-ray crystallography, providing structural insight into their catalytic flexibility.

## 1. Introduction

Lytic polysaccharide monooxygenases (LPMOs) are a distinct class of copper-dependent enzymes that have revolutionized our understanding of the enzymatic breakdown of polysaccharides (Bissaro *et al.*, 2018 ▸; Ipsen *et al.*, 2021 ▸; Johansen, 2016 ▸). These enzymes play a crucial role in the biodegradation of recalcitrant polysaccharides such as cellulose and chitin, thereby contributing significantly to the carbon cycle in nature (Johansen, 2016 ▸; Agger *et al.*, 2014 ▸; Hemsworth *et al.*, 2015 ▸; Vaaje-Kolstad *et al.*, 2010 ▸). LPMOs operate by an oxidative mechanism, which involves reduction of the active-site Cu(II) to Cu(I) and the cleavage of glycosidic bonds, a process that is essential for the efficient conversion of biomass in industrial applications (Kumar *et al.*, 2024 ▸; Chylenski *et al.*, 2019 ▸; Hemsworth *et al.*, 2015 ▸; Quinlan *et al.*, 2011 ▸; Johansen, 2016 ▸). In nature several reductants are possible, including proteins and small molecules, while ascorbic acid is one of the most commonly used reductants in the laboratory. LPMOs were initially grouped under glycoside hydrolases (GHs) and carbohydrate-binding modules (CBMs) (Liu *et al.*, 2007 ▸; Karkehabadi *et al.*, 2008 ▸). However, with advances in understanding their structure and function, LPMOs have been reclassified into auxiliary activity families AA9–AA11 and AA13–AA18 (Eijsink *et al.*, 2019 ▸; Rieder & Sørlie, 2023 ▸; Santos *et al.*, 2025 ▸) within the CAZy database. This reclassification reflects their unique oxidative mechanisms and the variety of substrates they act upon, including starch, cellulose, hemicellulose, chitin and pectin (Sabbadin *et al.*, 2021 ▸; Moreau *et al.*, 2019 ▸; Agger *et al.*, 2014 ▸).

Family AA9 comprises primarily (but not uniquely) fungal enzymes in plant cell-wall-degrading ascomycetes and basidiomycetes, aligning with their role in cellulose degradation, and has been intensively studied in terms of structure and function (Frandsen *et al.*, 2016 ▸; Zhang, 2020 ▸; Tandrup *et al.*, 2020 ▸; Batka *et al.*, 2024 ▸; Rieder *et al.*, 2021 ▸; Brander *et al.*, 2021 ▸).

LsAA9A, derived from *Lentinus similis* and classified within the AA9 family, is a widely used model for studying LPMOs due to its high-resolution crystal structures and its ability to bind to soluble oligosaccharides (Frandsen *et al.*, 2016 ▸, 2017 ▸). A notable focus has been the reported variation in the distance between the copper ion and the coordinating tyrosine residue (Tyr164), which some studies suggest may be linked to the enzyme’s redox state or substrate binding (Tandrup *et al.*, 2022 ▸). Nonetheless, the significance of these reported distance variations remains under debate, with conflicting interpretations (Walton & Davies, 2022 ▸).

Beyond the context of LPMO enzymology, this issue exemplifies a broader and fundamental challenge in structural biology: accurately capturing redox-sensitive metal sites under X-ray crystallographic conditions. A major complicating factor is photoreduction, X-ray radiation-induced reduction of the metal center, which can modify the coordination geometry and alter metal–ligand bond distances (Gudmundsson *et al.*, 2014 ▸; Yang *et al.*, 2011 ▸), but at the same time also replicate features of the biologically relevant catalytic cycles. Since the detected changes in geometry during redox cycling, by chemically induced reduction or X-ray photoreduction, can be very small, the issue of how well the bond lengths and angles can be measured is of great importance. As such, understanding the catalytically relevant geometry of LPMO active sites is not only critical for elucidating the catalytic mechanism but also serves as a case study in the limitations and interpretation of metalloprotein crystallography.

Accuracy describes how close a measurement is to the true or accepted value, whereas precision reflects its reproducibility, or how closely repeated measurements agree regardless of their accuracy. In structural biology, the relevant concept is coordinate precision; that is, the reliability with which atomic positions can be determined from diffraction data. For enzymes such as LPMOs, assessing coordinate precision is particularly challenging.

In small-molecule crystallography, coordinate precision is routinely derived from full-matrix least-squares refinement, which enables the calculation of standard uncertainties (s.u.s) for atomic positions (Agarwal, 1978 ▸; Templeton, 1999 ▸). These estimates are grounded in statistically well defined error models and are supported by the typically high data-to-parameter ratio of small-molecule datasets. As such, s.u. values are widely regarded as robust indicators of coordinate precision.

In macromolecular crystallography, however, a number of factors complicate the application of this direct approach. The large volumes of disordered solvent, conformational variability and frequent data incompleteness render full error propagation computationally impractical and statistically unreliable. Consequently, standard uncertainties are rarely reported in macromolecular structure determinations (Cruickshank, 1999 ▸). Instead, coordinate precision in macromolecular models is typically assessed through indirect means. Atomic displacement parameters (ADPs; Carugo, 2018 ▸), commonly expressed as *B* factors, offer qualitative insight into positional uncertainty, although they incorporate contributions from thermal motion, static disorder and model bias, and thus cannot be interpreted as direct measures of coordinate precision. Additional tools such as the Luzzati plot are also a significant method for estimating the precision of atomic positions (Luzzati, 1952 ▸). It allows an estimation of the upper limit of error in atomic coordinates by comparing the observed and calculated structure amplitudes at different resolution ranges, but it provides limited insight into local variations in coordinate precision. The diffraction precision index (DPI) introduced by Cruickshank (1999 ▸) offers an empirical approximation of mean coordinate error based on resolution, model *R* factor and the number of refined parameters. The DPI method allows estimation of coordinate errors not only at the global level but also for individual atoms and specific bond lengths (Kumar *et al.*, 2015 ▸). The accuracy of the DPI is highly resolution-dependent: it is considered most reliable at high resolution (∼1.0–1.5 Å), but at lower resolution (>2.5–3.0 Å) it often underestimates the true coordinate errors. These methods, among others, often form the basis for estimating coordinate precision in macromolecular individual crystal structure determination and are crucial for ensuring the reliability of the structural models produced.

In our previous work (Frandsen *et al.*, 2016 ▸) we detected a shortening of the Tyr O^η^ to copper bond length in the Cu(II) state of LsAA9A. In further work (Tandrup *et al.*, 2022 ▸), a difference of around 0.2 Å was found between the Cu(II) LsAA9A structures without and with bound cellotriose. By treating each structure as an independent estimate and comparing averages across multiple saccharide-free and saccharide-bound datasets, we observed a consistent difference in the Cu–Tyr O^η^ distance, indicating that the length estimate precision may exceed what could be expected from analysis based on DPI-based coordinate uncertainty alone. To exclude refinement artifacts, we computed *F*_o_ − *F*_c_ difference maps after rigid-body refinement using a saccharide-free model with fixed Cu–Tyr geometry against saccharide-bound data; the resulting difference density explicitly requires a copper shift towards Tyr O^η^, independently corroborating a genuine shortening of the Cu–Tyr bond. This trend is also reproduced qualitatively in QM/MM-optimized LsAA9A structures, where Cu–Tyr O^η^ decreases by ∼0.2–0.3 Å upon substrate binding (Theibich *et al.*, 2021 ▸). This difference was critically discussed in Walton & Davies (2022 ▸), who questioned the reliability of distance variations derived from single-crystal structures. In their analysis of multiple oxidation and substrate-bound states of the same AA9 LPMO, they applied a method-of-moments analysis to estimate global coordinate errors from pairwise atomic deviations. Based on this approach, they argued that the previously reported shortening of the Cu–Tyr O^η^ distance upon substrate binding falls within the estimated coordinate uncertainty and therefore should not be considered statistically significant.

Thus, on one hand we have statistical approaches estimating errors in geometric parameters based on individual crystal structures, suggesting relatively large errors in coordinates that preclude the assignment of statistical significance to the small geometric changes detected, and on the other hand our previous work suggesting that these geometric parameters are more reproducible across similar structures than would be expected from, for example, DPI estimates. In the present study, we delve further into this and systematically adopt a multiple structure-determination approach for distinct LPMO states to investigate the repeatability/reproducibility of distances and angles estimated from individual structures, where we treat the geometric parameters as direct measurements. Unlike in previous work (Tandrup *et al.*, 2022 ▸) all structure groups here are from the same protein batches and crystallization conditions, removing this source of variability across structures.

Recent computational studies have emphasized that the reduced Cu(I) state is essential for catalysis, as the priming reduction brings occupied copper *d* orbitals into the frontier energy region where they can engage in electron transfer (Wieduwilt *et al.*, 2024 ▸). Substrate binding, in contrast, appears to have a more subtle effect, primarily by shortening the Cu–Tyr O^η^ distance and modulating the alignment of co-substrate orbitals, while the tyrosine orbital itself remains largely nonreactive. To further investigate the structural effects of photoreduction, we extended our structural approach to chemically reduced LsAA9A crystals in both substrate-free and substrate-bound states, allowing the direct comparison of Cu(I) and Cu(II) geometries at 100 K and low X-ray dose, and the effect of high X-ray dose after chemical reduction was additionally investigated. Complementary to a previous study (Tandrup *et al.*, 2022 ▸), crystallographic studies were carried out at room temperature, where the structure is more flexible compared with the static conformation observed under cryogenic conditions. In this experiment, data were collected initially from the Cu(II) form and continued until X-ray-induced photoreduction to Cu(I) was expected to occur. The motivation for this study was to assess whether the structural effects of X-ray-induced reduction observed at cryogenic temperature are the same at a temperature where the enzyme can be catalytically active. We used a multi-crystal approach to overcome previously encountered issues due to global radiation damage, but – as before – could not obtain a fully photoreduced state at high resolution at room temperature, although we obtained a highly reliable room-temperature structure at 1.75 Å resolution with very little sign of photoreduction.

## 2. Materials and methods

### 2.1. Protein production and crystallization

LsAA9A was produced recombinantly in *Escherichia coli* and purified as described in Brander *et al.* (2021 ▸). Note that previous crystallographic work on this protein has also been carried out on fungally produced protein, which is glycosylated and methylated on His1 (Tandrup *et al.*, 2022 ▸). These modifications are absent in the protein produced recombinantly in *E. coli*. The protein concentration was initially 1.16 mg ml^−1^ in 20 m*M* sodium acetate pH 5.5, 150 m*M* sodium chloride. The protein stock solution was concentrated with an Amicon Ultra-15 centrifugal filter (3 kDa, Merck Millipore, Darmstadt, Germany) and the protein concentration was calculated from the measured *A*_280 nm_ (using a NanoDrop spectrophotometer) using the theoretical absorption coefficient determined from the sequence by ExPASy *ProtParam*. For active-site copper incorporation, equimolar copper(II) acetate from a 50 m*M* stock was added to the protein and incubated for one hour at refrigeration temperature for all of our crystallization experiments.

Crystallization was conducted using the hanging-drop vapor-diffusion method at room temperature in VDX plates (Hampton Research) with protein concentrations ranging from 2.0 to 6.5 mg ml^−1^. The reservoir solutions consisted of 1.4–2.2 *M* ammonium sulfate, 0.1 *M* sodium acetate buffer pH 4.5, with each well containing 500 µl reservoir solution and each drop consisting of 2 µl protein solution and 2 µl reservoir solution. Alternatively, crystallization of LsAA9A was performed using the sitting-drop vapor-diffusion method at room temperature in an MRC-Maxi (SWISSCI) with the protein concentration maintained at 1.16 mg ml^−1^. The reservoir solution consisted of 1.0–2.0 *M* ammonium sulfate, 0.1 *M* sodium acetate buffer pH 4.5, with each well containing 200 µl reservoir solution. Drops were set up by mixing 1 µl protein solution with 1 µl of the respective reservoir solution.

Crystals were prepared slightly differently for the dose-resolved study of photoreduction at room temperature. First, the protein buffer was exchanged to 25 m*M* sodium acetate pH 5.5 containing 20 m*M* sodium chloride. Subsequently, an equimolar solution of copper(II) acetate was added and the protein was soaked for 1 h at refrigeration temperature. Crystallization was carried out using the hanging-drop vapor-diffusion method at room temperature in VDX plates (Hampton Research). The protein concentration was adjusted to 1.16 mg ml^−1^ and each drop consisted of 1.5 µl protein solution mixed with 1.5 µl reservoir solution. The reservoir contained 1.4 *M* ammonium sulfate, 0.1 *M* sodium acetate buffer pH 4.5.

### 2.2. Data collection and structure determination

#### 2.2.1. 100 K single-crystal datasets of LsAA9A in Cu(II) and Cu(I) states (saccharide-free and saccharide-bound)

Structure labels used throughout the manuscript indicate the copper oxidation state, ligand condition and absorbed X-ray dose. Cell3 denotes cellotriose-bound structures, LD and HD denote low and high absorbed X-ray dose, respectively, and RT denotes room-temperature data collection. Accordingly, LsAA9A_Cu(II) and LsAA9A_Cu(I) refer to LsAA9A structures in the Cu(II) and Cu(I) states, respectively. LsAA9A_Cu(II)_LD, LsAA9A_Cu(II)_Cell3_LD, LsAA9A_Cu(I)_LD and LsAA9A_Cu(I)_Cell3_LD refer to low-dose structures in the corresponding copper oxidation and ligand states. LsAA9A_Cu(I)_HD and LsAA9A_Cu(I)_Cell3_HD refer to high-dose Cu(I) structures without and with bound Cell3, respectively. LsAA9A_RT_1.5kGy and LsAA9A_RT_88.5kGy refer to room-temperature structures collected with cumulative absorbed doses of approximately 1.5 and 88.5 kGy, respectively.

LsAA9A single crystals were harvested directly from the crystallization drops [LsAA9A_Cu(II)_LD] or soaked in a 0.5 *M* cellotriose (Cell3) solution containing mother liquor for 5 min [LsAA9A_Cu(II)_Cell3_LD]. For the chemically reduced experiment, crystals were first soaked in 10 m*M* ascorbic acid (Asc) as a reductant for 5 min and harvested [LsAA9A_Cu(I)_LD]. Subsequently, a subset of crystals were soaked in 0.5 *M* Cell3 drops for an additional 5 min [LsAA9A_Cu(I)_Cell3_LD]. Each crystal was mounted on a cryo-loop (Hampton Research) and flash-cooled. The absorbed X-ray dose reported for the ESRF data sets is the estimate from the *MXCuBE* interface based on the strategy program *BEST* (Bourenkov & Popov, 2006 ▸), which uses *RADDOSE* (Murray *et al.*, 2004 ▸) for calculation, and dose for the MAX IV data sets was calculated using the expected crystal lifetime calculator (https://bl831.als.lbl.gov/xtallife.html; Holton, 2009 ▸). X-ray diffraction data for LsAA9A and complex crystals were collected on the ID23-2 beamline at the European Synchrotron Radiation Facility (ESRF) in Grenoble, France and the BioMAX beamline at the MAX IV synchrotron in Lund, Sweden. The wavelengths used were 0.87 Å at ESRF and 0.98 Å at MAX IV. Data collection was performed with a transmission range of 0.1–0.2% at an exposure time of 10 ms for each image, ensuring a relatively low X-ray dose (below 10^5^ Gy) over 360° with a 0.1° oscillation. Furthermore, some of the crystals soaked in ascorbic acid solution (with or without Cell3) were collected at 10% transmission, yielding high-dose datasets. All datasets were processed using the *autoPROC* and *grenadesparallelproc* pipeline available at the beamlines, if necessary applying a stricter resolution cutoff using *XSCALE* (Kabsch, 2010 ▸). Data-processing statistics are given in Tables 1 ▸ and 2 ▸ and Supplementary Table S4.

The structures of LsAA9A_Cu(II) (PDB entry 7pyl) and LsAA9A_Cu(I) (PDB entry 7pqr) previously determined in our laboratory in space group *P*4_1_ were used as starting structures for refinement in all cases since all the LsAA9A saccharide-free and saccharide-complex crystals analyzed here were found to be isomorphous with this reference structure. Due to the characteristics of the *P*4_1_ space group, which allows two non-equivalent indexing orientations, re-indexing was performed using the *CCP*4 suite (Winn *et al.*, 2011 ▸; Agirre *et al.*, 2023 ▸) to ensure consistent indexing, if necessary, converting the index form *hkl* to *kh*−*l*. Subsequently, the *R*_free_ set was imported from the reference .mtz file, so that the same cross-validation set was used in all cases. The structures were solved by direct refinement using the saccharide-free models PDB entries 7pyl and 7pqr as the initial references for the Cu(II) and Cu(I) datasets, respectively. Prior to refinement, the copper ion as well as water and other nonprotein ligands were removed from the reference PDB entry to minimize model bias in the active site. Refinement was then carried out in *REFMAC*5 (Murshudov *et al.*, 1997 ▸) from the *CCP*4 suite, with subsequent model building in *Coot* (Emsley & Cowtan, 2004 ▸). For the dataset at 1.06 Å resolution, anisotropic refinement was applied at the final stage. Protein structure validation was performed by *PROCHECK* (Vaguine *et al.*, 1999 ▸) and full validation reports were generated by the wwPDB validation server. Ramachandran plot statistics were obtained using *RAMPAGE* (Lovell *et al.*, 2003 ▸) in the *CCP*4 suite. Refinement statistics are given in Tables 1 ▸ and 2 ▸ and Supplementary Table S4. Electron-density map figures were prepared using *PyMOL* (version 3.1.5.1; Schrödinger).

Based on these structures, copper–ligand coordination distances and angles were calculated. For each coordination parameter, a one-way ANOVA was performed across the four groups [LsAA9A_Cu(II)_LD, LsAA9A_Cu(II)_Cell3_LD, LsAA9A_Cu(I)_LD and LsAA9A_Cu(I)_Cell3_LD] to test whether the group means differed (*F* test comparing between-group with within-group variance). For parameters with ANOVA *p* < 0.05, we carried out Tukey–Kramer *post hoc* multiple comparisons to identify which group pairs differed, using the Tukey-adjusted significance threshold α = 0.05. The Tukey–Kramer procedure was chosen because it controls for multiple testing and accommodates unequal group sizes. All statistical analyses were performed in *Microsoft Excel* (version 16.106). As in structural biology one might in some cases just compare two states only (as here when we compare copper distances to the axial ligand), we also carried out pairwise comparisons using a *t*-test.

#### 2.2.2. Room-temperature multi-crystal datasets of substrate-free LsAA9A

A multi-crystal strategy was used to investigate photoreduction effects at room temperature by carrying out parallel data collections on several single crystals. To prevent dehydration during data collection, each crystal was mounted on a MicroMount loop and sealed with a MicroRT capillary (MiTeGen) containing 40 µl mother liquor. All mounting procedures were carried out in a humidity-controlled chamber (SLEEC, MiTeGen) with the relative humidity stabilized at approximately 97%. Diffraction data were collected at room temperature on MicroMAX at the MAX IV synchrotron, Lund, Sweden.

The X-ray beam was focused with compound refractive lenses, and the final beam size was defined by a 50 µm aperture positioned immediately upstream of the sample, resulting in a near-top-hat intensity profile matched to the ∼50 µm crystal dimensions. The beam flux at the sample position was measured with a photodiode (Hamamatsu). Two experiments were carried out. For the first experiment, where X-ray diffraction data were collected at a wavelength of 0.9544 Å, with the absorbed dose per image controlled at approximately 0.3 kGy and an oscillation range of 1° per image, 300 images were collected from 13 protein crystals. In the second experiment, at the same wavelength, data were collected from 12 protein crystals at approximately 0.6 kGy and an oscillation range of 1° per image. A total of 1440 images were collected for each crystal. The dose was estimated using the expected crystal lifetime calculator (https://bl831.als.lbl.gov/xtallife.html; Holton, 2009 ▸).

To enable dose-resolved analysis, in the first multi-crystal experiment dose effects were assessed by comparing two dose endpoints within each dataset. Specifically, the first and last ten images of the dataset (images 1–10 and 291–300) were processed, corresponding to cumulative absorbed doses of approximately 1.5 and 88.5 kGy, respectively. To ensure a consistent indexing scheme across all datasets, the first ten images from the dataset Sample 13 with the best overall data quality were processed independently and used as a reference for indexing. All remaining datasets were then processed using this reference. Each image subset was processed with *XDS* (Kabsch, 2010 ▸) and the resulting reflection files were scaled and merged using *XSCALE* (Kabsch, 2010 ▸). High-quality merged datasets were obtained from nine samples at 1.5 kGy (images 1–10) and seven samples at 88.5 kGy (images 291–300), with pairwise correlation coefficients between crystals ranging from 0.93 to 0.97. The merged .hkl files were subsequently converted to .mtz format. Because we did not observe copper reduction in this first experiment, we collected a second multi-crystal series with more images per dataset to extend the dose range.

In the second multi-crystal experiment, each dataset was divided into several subsets of 50 images. The first 50 images from Sample 11, which exhibited the best overall data quality, were used as the indexing reference. Based on scaling statistics and completeness, three samples were selected for further analysis. For each of these samples, subsets 1–50, 351–400, 651–700 and 951–1000 were processed, representing cumulative absorbed doses of approximately 15, 225, 405 and 585 kGy, respectively. Data processing and scaling followed the same procedure as described above. The merged datasets showed pairwise correlation coefficients exceeding 0.8 and were converted to .mtz format. Subsequent structure determination and refinement were performed as described above. Data-processing and refinement statistics are provided in Supplementary Tables S6 and S8.

DPI was calculated for all protein structures in this study. DPI values [σ(*r*)] were computed using the Cruickshank formulation (Cruickshank, 1999 ▸):
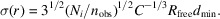
*N_i_* is the number of independently refined parameters in the model and *n*_obs_ is the number of unique observed reflections included in refinement. *C* is the fractional completeness of the data to *d*_min_, which is the high-resolution limit of the dataset, and *R*_free_ is the cross-validation residual. We report the *R*_free_-based DPI as the estimated positional error.

## 3. Results and discussion

### 3.1. Overview and preliminary structural analysis of the Cu(II) and Cu(I) states at 100 K

**Figure 1:**
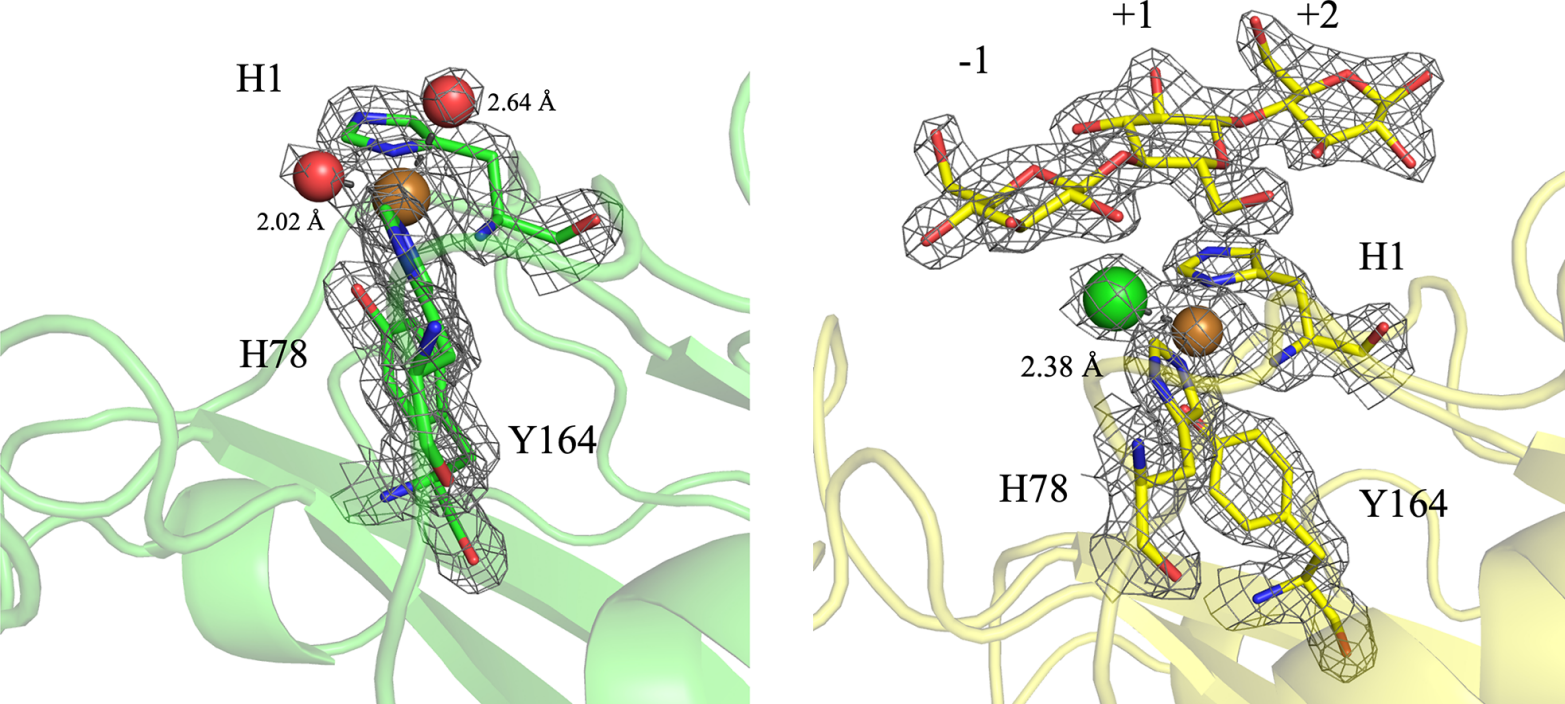
Electron-density map of the copper active site from LsAA9A_Cu(II)_LD_9tcv (left) and LsAA9A_Cu(II)_Cell3_LD_9td1 (right). In LsAA9A_Cu(II)_LD_9tcv one equatorial water molecule is coordinated to the copper at a distance of 2.02 Å, while the axial water distance is 2.64 Å. Sulfate and an additional water, each with 0.5 occupancy, are within hydrogen-bonding distance of the equatorial water but are not shown here for simplicity (shown in Supplementary Fig. S1). In LsAA9A_Cu(II)_Cell3_LD_9td1, the equatorial Cl^−^ ligand is positioned at a distance of 2.38 Å from the copper. 2*F*_o_ − *F*_c_ electron density is shown at a 1.0σ contour level as a gray mesh. The brown sphere is copper, red spheres are H_2_O and the green sphere is Cl^−^.

Data and refinement statistics and accession codes for LsAA9A_Cu(II)_LD and LsAA9A_Cu(II)_Cell3_LD structures at maximum resolutions between 1.6 and 2.0 Å are shown in Table 1 ▸. As shown in the electron-density maps in Fig. 1 ▸ and Supplementary Fig. S1 for LsAA9A_Cu(II)_LD_9tcx, there are two distinct waters at equatorial and axial positions consistent with a primarily Cu(II) state, according to previous studies (PDB entry 7pyl; Tandrup *et al.*, 2022 ▸) on the same enzyme at a low dose of 1.49 × 10^4^ Gy, where the distances from the copper were 1.94 Å for the equatorial water and 2.63 Å for the axial water. The availability of four similarly determined structures (three presented here and PDB entry 7pyl) allows us to estimate the standard deviation of the measured bond distances (Supplementary Table S1) by treating them as direct measurements. As previously (Tandrup *et al.*, 2022 ▸), we observe that the reproducibility of distance estimates is much higher than suggested by the coordinate precision estimated by DPIs (see, for example, Tables 1 ▸ and 2 ▸, showing an average DPI of 0.23 Å). Even considering that some of the involved atoms could have atomic DPIs that are significantly below average, a propagation-of-error approach would give an error on bond length of [(DPI − atom1)^2^ + (DPI − atom2)^2^]^1/2^, which would be 0.28 Å with DPIs of 0.2 Å and 0.14 Å for DPIs of 0.1 Å. Yet for the vast majority of the interatomic distances analyzed in Supplementary Tables S1 and S2, with the exception of some of the distances involving exogenous ligands, the deviation of individual measurements from the average is below the DPI-derived uncertainties.

While a detailed discussion of the reasons for this discrepancy is beyond the scope of this article, especially since we cannot generalize from the specific LsAA9A case without further experimental data, we suggest that the coordinate uncertainties determined by DPIs incorporate effects of lattice disorder and imperfection, and a propagation-of-error approach treats the individual atom positions in an interacting pair as uncorrelated. This assumption may overestimate the calculated uncertainties on interatomic distances.

For LsAA9A_Cu(II)_LD structures, the corresponding copper-to-equatorial water and copper-to-axial water distances were found to be 1.99 ± 0.10 and 2.69 ± 0.06 Å, respectively. In the LsAA9A_Cu(II)_Cell3_LD structure, the axial water is displaced by the saccharide (Fig. 1 ▸ for PDB entry 9td1). Loss of water on saccharide binding is probably a general feature of LPMOs and has also recently been demonstrated by spectroscopic analysis of a chitin-active LPMO (Joseph *et al.*, 2025 ▸). As in previous work (Tandrup *et al.*, 2022 ▸), Cell3 occupies subsites −1 to +2, where the numbering follows the standard convention, with negative numbers indicating subsites towards the nonreducing end and positive numbers those towards the reducing end of the substrate, and chloride, derived from the protein-storage buffer, was modeled at the equatorial position as before (Fig. 1 ▸ and Supplementary Fig. S1). Although the data collection was not optimized to obtain anomalous signal, low-level anomalous difference density was observed in one of the three triplicate structures at the Cl^−^ position, further confirming that this electron-density peak must be interpreted as Cl^−^ and not water. The Cl^−^ distance (Supplementary Table S1) is very similar as in the previous low-dose LsAA9A_Cu(II)_Cell3 (PDB entry 7pyu), with a final estimate of 2.42 ± 0.0903 Å. Therefore, the geometry indicates that the copper centers in our LsAA9A_Cu(II)_Cell3_LD structures are primarily in the Cu(II) oxidation state.

**Figure 2:**
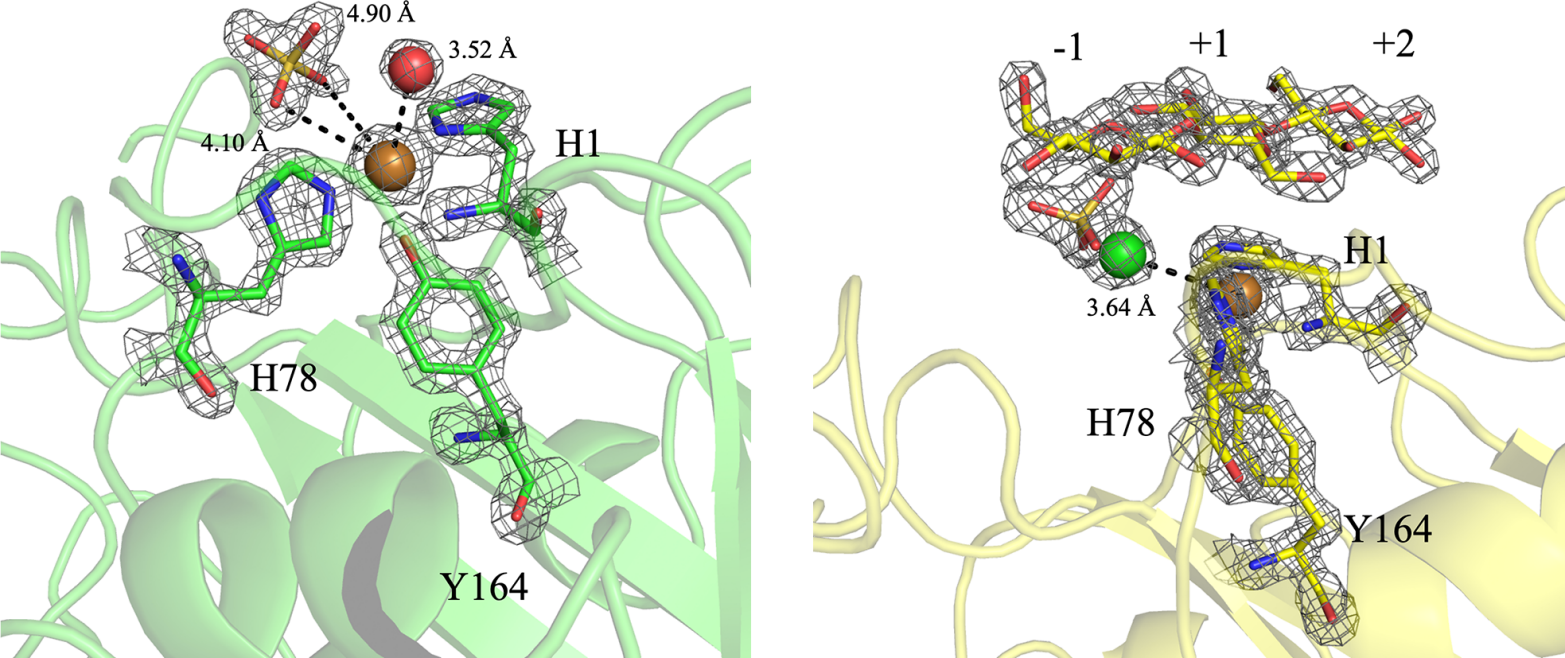
Electron-density map of the copper active site from LsAA9A_Cu(I)_LD_9td9 (left) and LsAA9A_Cu(I)_Cell3_LD_9tdc (right). In LsAA9A_Cu(I)_LD_9td9 one axial water molecule is coordinated to the copper at a distance of 3.52 Å and a sulfate with 0.6 occupancy is in an equatorial position with a distance of around 4.10 Å. In LsAA9A_Cu(I)_Cell3_LD_9tdc, at the equatorial position there is a Cl^−^ ion with 0.5 occupancy and a sulfate ion with 0.5 occupancy. The equatorial Cl^−^ ligand distance to the copper is 3.64 Å. 2*F*_o_ − *F*_c_ electron density is shown at a 1.0σ contour level as a gray mesh. The brown sphere is copper, the red sphere is H_2_O and the green sphere is Cl^−^.

Crystal structures of LsAA9A soaked in ascorbic acid (as a reductant) solutions, both in the presence and absence of Cell3, were determined (Table 2 ▸) at a comparable dose to the previous data sets for the Cu(II) structures. As shown in the electron-density maps in Fig. 2 ▸ and Supplementary Fig. S1 for LsAA9A_Cu(I)_LD_9td9, there is a clear water at the axial position with a distance of 3.52 Å, and the average distance from three structures is 3.43 ± 0.09 Å. Compared with the previously reported ligand distance of 3.77 Å in the Cu(I) state (PDB entry 7pyq; Tandrup *et al.*, 2022 ▸), our structures here are in the Cu(I) state. In previous work (Tandrup *et al.*, 2022 ▸), the ascorbic acid-reduced fungal protein structures also show similar copper axial water ligand distances of 3.23 Å at low dose (PDB entry 7pxu) and 3.49 Å at high dose (PDB entry 7pxv). At the equatorial position, a sulfate is located approximately 4.0 Å away (Fig. 2 ▸, left). In the structure LsAA9A_Cu(I)_LD_9td9, the sulfate is modeled with an occupancy of around 0.6, while in the other two structures it is modeled as a mixed site with 0.5 sulfate ion and 0.5 water. In the Cell3-bound structures, the presence of Cell3 at the axial position is accompanied by a Cl^−^ ion with 0.5 occupancy and sulfate ion with 0.5 occupancy at the equatorial position, as shown in Fig. 2 ▸ and Supplementary Fig. S1 for LsAA9A_Cu(I)_Cell3_LD_9tdc. The Cl^−^ distance of 3.64 Å in this structure is consistent with our measurements, which gave an average Cl^−^ distance of 3.50 ± 0.25 Å across the structures analysed here, and the reported distances of 3.83 Å in the LsAA9A_Cu(I)_Cell3 structure (PDB entry 7pz0; Tandrup *et al.*, 2022 ▸) and 3.83 Å in the related structure (different crystal form) obtained with fungally produced LsAA9A (PDB entry 7pyi), supporting the presence of a chloride ligand at the equatorial site but out of coordination distance. In at least one of our triplicates, the anomalous difference map supports the presence of two anomalously scattering species, with excellent density confirming that one of them is a sulfate ion.

### 3.2. Statistical comparison of the Cu(II) and Cu(I) states using an ANOVA test

As detailed in Section 1, we have previously (Tandrup *et al.*, 2022 ▸) reported a consistent reduction in the Tyr O^η^ to Cu distance for LsAA9A upon binding with saccharide substrates; however, due to the small magnitude of the changes, others have questioned the significance of the results. Here, we systematically investigate the reproducibility across multiple structure determinations under comparable conditions, using chemical reduction to achieve the Cu(I) states.

Geometric parameters were measured in the four groups of structures representing Cu(II)/Cu(I) states with/without saccharide, and included distances between the active-site copper and coordinating atoms (His1 N^δ1^, His1 N^Am^, His78 N^ɛ2^ and Tyr O^η^), the bond angles θ_1_, θ_2_ and θ_3_ (defined by atoms His1 N^δ1^–Cu–His1 N^Am^, His1 N^Am^–Cu–His78 N^ɛ2^ and His1 N^δ1^–Cu–His78 N^ɛ2^, respectively) and the θ_T_ angle between the His1 N^δ1^–Cu–His1 N^Am^ plane and the His78 N^ɛ2^–Cu line (Supplementary Tables S1 and S2).

One-way ANOVA tests were performed with degrees of freedom of 3 and 10 for the between-group and within-group variations, respectively (Table 3 ▸). The results indicate that Cu–His1 N^δ1^ (*p* = 0.04010), Cu–Tyr O^η^ (*p* < 0.00043), θ_2_ (*p* < 0.00012), θ_3_ (*p* = 0.00662) and θ_T_ (*p* = 0.00340) show statistically significant differences between groups. In contrast, Cu–His1 N^Am^ (*p* = 0.07579), Cu–His78 N^ɛ2^ (*p* = 0.18111) and θ_1_ (*p* = 0.28028) do not display significant variation between groups. These results suggest that the Cu–His1 N^δ1^ and Cu–Tyr O^η^ bond lengths and certain angular parameters (θ_2_, θ_3_ and θ_T_) exhibit measurable variation across experimental conditions.

### 3.3. Tukey–Kramer *post hoc* test

**Figure 3:**
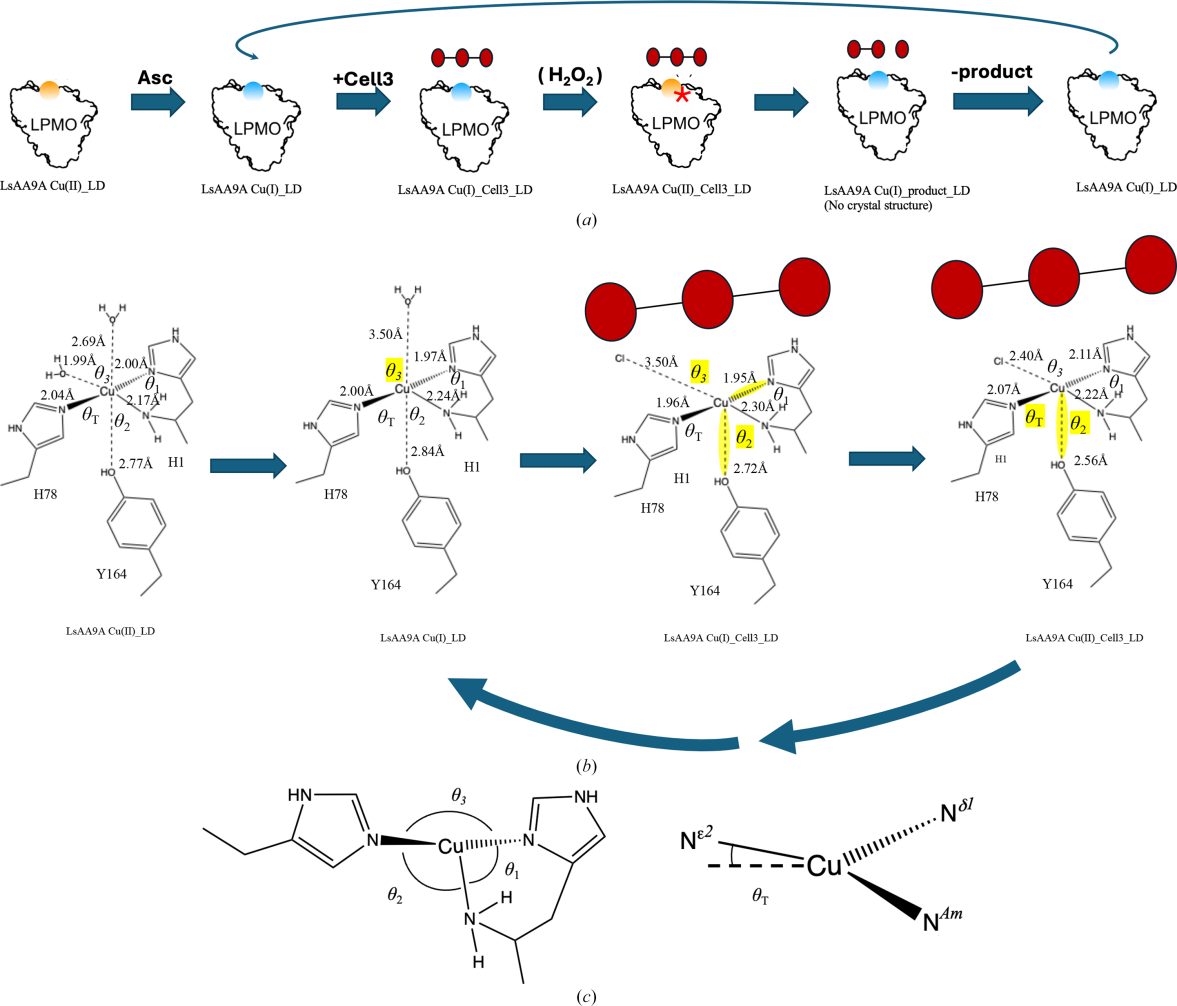
(*a*) Reconstructed catalytic cycle of LsAA9A with Cell3 as substrate and H_2_O_2_ as co-substrate. The Cl^−^-bound structure represents an unreactive mimic of the H_2_O_2_-bound state (* means Cl^−^). The label H_2_O_2_ is shown in parentheses to indicate that hydrogen peroxide was not added experimentally. Cu(II)_H_2_O_2__substrate intermediates are likely to be too reactive to be captured by single-crystal crystallography. A product-bound structure is included but has not yet been trapped crystallographically. See the main text for further details. (*b*) Schematic representation of the active-site geometries of LsAA9A under four different conditions, Cu(II), Cu(I), Cu(I)–Cell3 and Cu(II)–Cell3, showing the mean distances of atoms coordinated to the copper center. The figure illustrates selected significant structural parameter differences according to the Tukey–Kramer test, highlighted in yellow. Only differences between states that can be considered to be sequential according to the scheme in (*a*) are highlighted: LsAA9A_Cu(II)_LD → LsAA9A_Cu(I)_LD → LsAA9A_Cu(I)_Cell3_LD → LsAA9A_Cu(II)_Cell3_LD [mimicking LsAA9A_Cu(II)_Cell3-HO—OH] →→ LsAA9A_Cu(I)_LD. From LsAA9A_Cu(I)_LD to LsAA9A_Cu(I)_Cell3_LD, θ_3_ changes significantly, decreasing from 168.27 ± 0.61° to 161.37 ± 1.89. The transition from LsAA9A_Cu(I)_Cell3_LD to LsAA9A_Cu(II)_Cell3_LD involves marked changes in θ_2_, θ_3_ and the Cu–His1 N^δ1^ and Cu–Tyr O^η^ distances: θ_2_ decreases from 100.56 ± 1.10° to 90.75 ± 2.03°, θ_3_ increases from 161.37 ± 1.89° to 166.90 ± 2.12°, the Cu–His1 N^δ1^ distance increases from 1.95 ± 0.03 Å to 2.11 ± 0.08 Å and the Cu–Tyr O^η^ distance decreases from 2.72 ± 0.06 Å to 2.56 ± 0.04 Å. Finally, the transition from LsAA9A_Cu(II)_Cell3_LD to LsAA9A_Cu(I)_LD involves marked changes in θ_2_, θ_T_ and the Cu–Tyr O^η^ distance: θ_2_ increases from 90.75 ± 2.03° to 97.92 ± 1.60°, θ_T_ decreases from 12.40 ± 2.07° to 4.90 ± 2.06° and the Cu–Tyr O^η^ distance increases from 2.56 ± 0.04 Å to 2.84 ± 0.08 Å. A similar illustration, highlighting significant differences according to pairwise *t*-tests, is shown in Supplementary Fig. S2. (*c*) Definition of θ_1_, θ_2_, θ_3_ and θ_T_. θ_1_–θ_3_ are the N—Cu—N bond angles as indicated. θ_T_ is the angle between the N^δ1^–Cu–N^Am^ plane and the N^ɛ2^–Cu vector.

Following the one-way ANOVA test, a Tukey–Kramer *post hoc* test was performed to assess differences among groups. Additionally, pairwise two-tailed *t*-test results were included to complement the Tukey–Kramer test, providing a reference for comparisons limited to two states (for example, axial water distances in the non-saccharide-bound states). The results are summarized in Supplementary Table S3, while selected results are shown in Fig. 3 ▸(*b*) (Tukey–Kramer test) and Supplementary Fig. S2 (*t*-test).

Among the copper–ligand coordination distances compared between the LsAA9A_Cu(II)_LD and LsAA9A_Cu(II)_Cell3_LD structures, only the Cu–Tyr O^η^ distance exhibited a statistically significant difference of approximately 0.2 Å. We also note that the average *B* factor for the Tyr O^η^ atom is lower than for other copper ligands in both set of structures; thus, the length difference is not a result of less precise coordinate determination. Across the two Cu(II) datasets, the *B* factors (mean ± SD) for the copper-coordinated atoms were 18.745 ± 7.829 (His1 N^δ1^), 19.755 ± 9.386 (His1 N^Am^), 18.830 ± 8.483 (His78 N^ɛ2^) and 16.343 ± 7.016 Å^2^ (Tyr O^η^) in LsAA9A_Cu(II)_LD; the corresponding values were 31.43 ± 14.47 (His1 N^δ1^), 27.33 ± 10.90 (His1 N^Am^), 27.24 ± 10.60 (His78 N^ɛ2^) and 24.67 ± 9.62 Å^2^ (Tyr O^η^) in LsAA9A_Cu(II)_Cell3_LD. The average Cu–Tyr O^η^ distance was 2.77 ± 0.0585 Å in LsAA9A_Cu(II)_LD and 2.56 ± 0.0377 Å in LsAA9A_Cu(II)_Cell3_LD [also significantly shorter compared with 2.84 ± 0.0794 Å in LsAA9A_Cu(I)_LD and 2.72 ± 0.0577 Å in LsAA9A_Cu(I)_Cell3_LD]. Therefore, we confirm that upon binding of saccharide substrate the Cu–Tyr O^η^ distance in LsAA9A is consistently diminished for the Cu(II) form. There is no statistically significant difference in the Cu–Tyr O^η^ distance between the LsAA9A_Cu(I)_LD and LsAA9A_Cu(I)_Cell3_LD structures or the saccharide-free Cu(II) and the Cu(I) state, similar to as suggested in our previous study in which the Cu(I) state was obtained by photoreduction (Tandrup *et al.*, 2022 ▸).

As shown in Supplementary Table S3, both the Tukey–Kramer test and the *p*-values for the distances involving His1 N^δ1^ and Tyr O^η^ (all below 0.05) indicate significant changes in the coordination environment upon soaking in ascorbic acid in the presence of Cell3.

Notably, in the statistical analysis of angles, the previously reported increase in θ_2_ and decrease in θ_3_ angles on photoreduction of Cu(II) to Cu(I) (Tandrup *et al.*, 2022 ▸) is most statistically significant in the saccharide-bound states of the chemically reduced LsAA9A crystals (Tukey–Kramer test and *t*-test). θ_2_ increases from 90.75 ± 2.0279° for LsAA9A_Cu(II)_Cell3_LD to 100.56 ± 1.1092° for LsAA9A_Cu(I)_Cell3_LD, and θ_3_ decreases from 166.90 ± 2.1190° for LsAA9A_Cu(II)_Cell3_LD to 161.37 ± 1.8937° for LsAA9A_Cu(I)_Cell3_LD. In the unbound state, only the increase in θ_2_ is statistically significant and only in the pairwise *t*-test.

In terms of the effect of saccharide binding, we see clear changes between bound and unbound states, the most statistically significant (Tukey–Kramer test) being an increase in θ_2_ when comparing the LsAA9A_Cu(II)_LD state with LsAA9A_Cu(I)_Cell3_LD, rising from 94.22 ± 1.8658° to 100.56 ± 1.1092°, and a decrease between LsAA9A_Cu(I)_LD and LsAA9A_Cu(II)_Cell3_LD from 97.92 ± 1.5974° to 90.75 ± 2.0279°. However, since the most significantly different structures also have a different redox state, it is difficult to ascertain the effect of bound substrate alone on the θ_2_ angle.

Moreover, θ_3_ decreases significantly (Tukey–Kramer test) on substrate binding in the Cu(I) state [168.27 ± 0.6106° for LsAA9A_Cu(I)_LD to 161.37 ± 1.8937° for LsAA9A_Cu(I)_Cell3_LD].

Loss of planarity at the copper site on substrate binding in both copper redox states is shown by the increased values of θ_T_ in LsAA9A_Cu(II)_Cell3_LD (12.40 ± 2.0702°), being significantly different from LsAA9A_Cu(II)_LD (5.41 ± 3.0266°) (Tukey–Kramer test), and LsAA9A_Cu(I)_Cell3_LD (10.22 ± 1.6256°), being significantly different from LsAA9A_Cu(I)_LD (4.90 ± 2.0561°) (*t*-test).

In addition to the geometric parameters showing significant differences in the ANOVA test, we carried out a pairwise comparison of axial ligand distances to the copper, which was not included in the ANOVA analysis due to the absence of axial ligands in the saccharide-bound states. The axial water molecule exhibits a mean distance of 3.43 ± 0.09 Å, compared with 2.69 ± 0.06 Å in the LsAA9A_Cu(II)_LD structures. This difference is statistically significant (*p* = 0.00004). We did not include equatorial ligands in the ANOVA statistical analysis due to the large uncertainties in some of the states, different ligands and sometimes difficulties in modeling the equatorial ligands due to a vicinal sulfate ion. However, a few general comments can be made. In the LsAA9A_Cu(II)_LD structures the equatorial distance between the coordinated H_2_O molecule and the copper active site averages 1.99 ± 0.10 Å. In contrast, in the LsAA9A_Cu(II)_Cell3_LD structures the corresponding equatorial distance involving a Cl^−^ ion averages 2.42 ± 0.09 Å. In the LsAA9A_Cu(I)_LD structures the equatorial water ligand is replaced by a sulfate.

Moreover, in the LsAA9A_Cu(I)_Cell3_LD structures a Cl^−^ ion occupies the equatorial position with an average distance of 3.50 ± 0.25 Å, whereas the corresponding distance in the LsAA9A_Cu(II)_Cell3_LD structures is significantly shorter (*p* = 0.00041).

### 3.4. A reconstructed catalytic cycle for the LPMO reaction

Based on the static structures at 100 K described above, we attempted to reconstruct the catalytic cycle of LsAA9A (Fig. 3 ▸*a*). In the initial priming step, the copper center at the active site is reduced from its oxidized Cu(II) state to the catalytically active Cu(I) form. The LsAA9A structures obtained at low X-ray dose without saccharide [denoted LsAA9A_Cu(II)_LD above] represent the state before priming, while the structure obtained under similar conditions but after the addition of ascorbic acid [LsAA9A_Cu(I)_LD structures described above] represents the state after priming. This reduction is essential to initiate the oxidative cleavage of polysaccharide substrates and has also been suggested to increase the affinity of LPMOs for saccharides (Kracher *et al.*, 2018 ▸; Christensen *et al.*, 2023 ▸; Brander *et al.*, 2021 ▸). Thus, it is reasonable to expect that during the catalytic cycle in solution, substrate binding occurs primarily in the reduced LPMO state, as we and others have also assumed (Christensen *et al.*, 2023 ▸; Brander *et al.*, 2021 ▸; Kracher *et al.*, 2018 ▸; Joseph *et al.*, 2025 ▸). Once the LPMO active-site metal is reduced, the saccharide (in our experiments represented by Cell3) binds to the active site, aligning the glycosidic bond for subsequent oxidation. This state can be represented by the LsAA9A_Cu(I)_Cell3_LD structures that we have described above. The reduced Cu(I)–substrate complex then reacts with the co-substrate H_2_O_2_, driving the hydroxylation and cleavage of the polysaccharide chain. As no H_2_O_2_ is added in our experiment, we do not have this exact state represented in our structure; furthermore, the species is expected to be very short-lived and not easily trapped in a static X-ray crystal structure. However, it was suggested in previous papers (Frandsen *et al.*, 2016 ▸) that chloride ions, which at high concentration are inhibitory (Di Domenico *et al.*, 2025 ▸), are an unreactive mimic of the co-substrate. Recent theoretical calculations have used previously published Cl^−^-bound structures to construct H_2_O_2_-bound reactive species (Wieduwilt *et al.*, 2024 ▸). Thus, we argue that our structure LsAA9A_Cu(II)_Cell3_LD with bound Cl^−^ likely represents the geometry of the LPMO just prior to the homolytic splitting of H_2_O_2_ [LsAA9A_Cu(II)_Cell3-HO—OH] and cleavage of the glycosidic bond. After the cleavage reaction, release of the saccharide product returns the enzyme to its Cu(I) state [LsAA9A_Cu(I)_LD].

Geometric parameters that differ significantly between structures are visualized according to this reconstructed path based on the Tukey–Kramer test in Fig. 3 ▸(*b*) and pairwise *t*-test in Supplementary Fig. S2. Compared with the Tukey–Kramer test, additional geometric parameters show significant differences in the pairwise *t*-test. Specifically, significant differences were observed for θ_2_ between LsAA9A_Cu(II)_LD and LsAA9A_Cu(I)_LD, for θ_T_ between LsAA9A_Cu(I)_LD and LsAA9A_Cu(I)_Cell3_LD, and for the Cu–His1 N^δ1^ distance between LsAA9A_Cu(II)_LD and LsAA9A_Cu(I)_LD in the *t*-test.

### 3.5. Additional photoreduction of Asc structures

To assess whether high X-ray doses induce additional changes to the geometry of the already chemically reduced LsAA9A_Cu(I) metal-binding site, crystals soaked in ascorbic acid, with and without Cell3, were measured under high-dose (∼10^3^ kGy) conditions. Two datasets were collected for each condition, and the corresponding data-collection and refinement statistics are summarized in Supplementary Table S4, showing reasonable *R*_work_ and *R*_free_ values. The distances and angles of coordinating atoms with the copper active site were measured and are presented in Supplementary Table S5, where they are compared with the average. From the results, across all parameters (Cu–His1 N^δ1^, Cu–His1 N^Am^, Cu–His78 N^ɛ2^, Cu–Tyr O^η^, θ_1_–θ_3_ and θ_T_), for the substrate-free Cu(I) structures the maximum absolute difference in mean bond lengths is 0.08 Å (Cu–His78 N^ɛ2^) and the largest angular deviation is 1.66° (θ_1_), and for the substrate-bound Cu(I) structures the corresponding maxima are 0.04 Å (Tyr O^η^) and 1.01° (θ_2_). These results indicate that the coordination geometry remains stable, suggesting that exposure to higher X-ray dose does not induce detectable geometric distortion at the active site for LsAA9A_Cu(I) with and without substrate bound. Interestingly, ascorbic acid has been reported to be a radical scavenger (Murray & Garman, 2002 ▸) and may help protect the active site. Significant negative electron-density features were though observed in the disulfide-bond region, providing direct evidence of additional site-specific radiation-induced damage, shown in Supplementary Fig. S3.

### 3.6. Photoreduction experiments at room temperature

A preliminary experiment at 1.5 and 88.5 kGy was carried out. To maintain an outer shell CC_1/2_ of approximately 40%, the resolution cutoffs were set to 1.75 Å for the 1.5 kGy dataset (PDB entry 9tde) and 1.85 Å for the 88.5 kGy dataset (PDB entry 9tdf). We expected that photoreduction of Cu(II) might be evident at a lower dose at room temperature compared with 100 K, but this was not the case. Little sign of reduction of Cu(II) was seen in terms of coordinating ligands, since the equatorial and axial water molecules were clearly visible at a distance consistent with Cu(II), shown in Supplementary Fig. S4. We were interested in seeing whether indications of photoreduction were present in other geometric changes. While our own Tukey–Kramer analysis does not detect significant structural differences between the Cu(II) and Cu(I) states without saccharide and a pairwise *t*-test highlights only θ_2_ changes as possibly statistically significant, previous large-scale analyses of LPMO geometry report an increase in θ_2_ and a decrease in θ_3_ (Vu & Ngo, 2018 ▸; Ciano *et al.*, 2018 ▸), changes that we also noted in our previous dose-dependent study of LsAA9A and TaAA9A (Tandrup *et al.*, 2022 ▸). These changes therefore, if not strongly statistically significant, are at least widely reported for several different structurally characterized LPMOs. It is thus interesting to note that while θ_1_ shows little change, θ_2_ increases from 91.16° to 96.25° and θ_3_ correspondingly decreases from 175.25° to 166.95° as exposure to X-rays increases from 1.5 to 88.5 kGy (Supplementary Table S7). In fact, as in the single-crystal room-temperature structure collected on an in-house source presented previously (Tandrup *et al.*, 2022 ▸), the θ_2_ (91.16°) is one of the lowest recorded for LsAA9A, while the θ_3_ (175.25°) is one of the highest recorded for the 1.5 kGy structure, suggesting these are the LsAA9A_Cu(II) structures least contaminated by Cu(I) generated by X-ray photoreduction.

In a second experiment, data sets representing snapshots at average doses of 15, 225, 405 and 585 kGy were taken. At increased doses, vastly reduced pairwise correlations between data sets indicated global damage. To maintain an acceptable pairwise correlation between data sets, the number of crystals merged was reduced to three (50 frames for each), retaining only those with consistent diffraction quality and high pairwise correlations over 0.8. Furthermore, a serious reduction of overall quality – a sign of global radiation damage – was seen. In order to maintain a CC_1/2_ of around 40% in the outer resolution shells, the following resolution limits were applied: 1.50 Å for the 15 kGy structure, 1.90 Å for the 225 kGy structure, 2.60 Å for the 405 kGy structure and 2.80 Å for 585 kGy structure. Data statistics are shown in Supplementary Table S8. As expected from the previous experiments, in the structures from initial datasets at 15 and 225 kGy doses, the axial and equatorial ligands are clearly visible in an initial difference map where these ligands are not modeled (Supplementary Fig. S5), demonstrating a large component of Cu(II). In the latter structures these are not clearly visible. However, this could be an effect of global radiation damage and lower resolution. To test this, all structures were refined from the same starting point without ligands, imposing the same resolution limit of 2.80 Å. Data-collection and structure-refinement statistics are shown in Supplementary Table S9. The electron densities show that at this resolution the position of the axial ligand is not very clear even in the 15 kGy structure (Supplementary Fig. S5). Furthermore, θ_2_ and θ_3_ do not follow the trends reported above in the refined structures at 2.80 Å (the measured values of θ_2_ and θ_3_ are 97.817°/171.343° at 15 kGy, 96.308°/172.623° at 225 kGy, 104.559°/166.048° at 405 kGy and 95.0°/169.9° at 585 kGy) for this second photoreduction experiment, also suggesting that the structures are not sufficiently reliable for this analysis. We see the global radiation damage and very different resolutions as a serious obstacle to appropriate geometric characterization and comparison of the active site. It seems that in practice it will not be possible to study a fully photoreduced copper site at room temperature, even using a multiple crystal approach, due to the general overall quality of data and global radiation damage to the structure occurring before the Cu(II) to Cu(I) transition has fully occurred, and thus we have chosen not to deposit the structures from the second photoreduction experiment. It is interesting to see though that the observed changes in θ_2_ and θ_3_ occur in the first photoreduction experiment, even before a loss of exogenous ligands is clearly visible in the electron-density maps.

## 4. Conclusions

For metalloproteins X-ray-induced photoreduction is common, and balancing X-ray dose with diffraction intensity is essential. In LPMOs, photoreduction of the active-site copper is difficult to avoid but can provide mechanistic insights into the initial catalytic step. Our multiple diffraction experiments confirm that primarily the Cu(II) state is maintained below a total dose of 50 kGy (Table 1 ▸), whereas the full Cu(I) state can be reliably generated at approximately 10^3^ kGy (Table 2 ▸) from previous work (Tandrup *et al.*, 2022 ▸). Accordingly, we recommend these dose thresholds for crystallographic studies of AA9 LPMOs, although they may well vary across different proteins and crystallization conditions.

Furthermore, the presented dose-dependent study at room temperature suggests that the doses required for full photoreduction may be similar at room temperature as they are at 100 K, since clear evidence of close equatorial and axial ligands is present in the 225 kGy structure (Supplementary Fig. S5), suggesting significant proportions of the Cu(II) state. However, a final conclusion cannot be reached on the basis of our current study, since at higher doses global radiation damage precludes analysis.

In parallel ongoing work, we are exploring serial crystallography as a means to follow LPMOs in action. Much time-resolved serial crystallography research uses light as a reaction trigger, but since LPMOs are not light-sensitive, and given the importance of the priming reaction, one possible avenue would be triggering by photoreduction. Here, we show that despite the implementation of a multi-crystal approach, global radiation damage remains an intrinsic limitation, and the diminished resolution obtained under high-dose conditions precludes resolving a high-resolution structure corresponding to a fully photoreduced Cu(I) state at room temperature. It might be possible to achieve a compromise between damage and active-site reduction at lower temperatures (for example 4–10°C), where partial reactivity could be preserved while mitigating radiation-induced effects. This will be the subject of future investigations. Currently, however, chemical reduction appears to be the most suitable method for inducing Cu(II) to Cu(I) conversion in LPMOs for mechanistic crystallographic studies, as it also potentially exploits the protective effect of ascorbic acid against radiation damage (Murray & Garman, 2002 ▸).

The present study clearly shows that the structural changes accompanying chemical reduction of the copper center at 100 K closely resemble those previously reported upon X-ray photoreduction and with chemical reduction in fungally produced LsAA9A without saccharide. Importantly, further X-ray exposure, while inducing additional site-specific radiation damage, shown for example by rupture of a disulfide bond (between Cys41 and Cys167) in the LsAA9A_Cu(I)_Cell3_HD structures in Supplementary Fig. S3, does not appear to alter the geometry of the active site compared with the LsAA9A_Cu(I)_Cell3_LD structures, suggesting that the reduced-state coordination environment is robust under these conditions (Supplementary Table S5).

By systematically comparing the Cu(II) and Cu(I) states with and without substrate bound at 100 K, a LPMO reaction pathway was reconstructed (Fig. 3 ▸*a*), in which we identified which geometric parameters undergo statistically significant changes (Fig. 3 ▸*b*, Supplementary Fig. S2 and Supplementary Table S3). It is somewhat surprising that the Tukey–Kramer test only detects significant changes during the priming reduction to Cu(I) in the saccharide-bound state, despite many previous reports of systematic changes in the unbound form (Ciano *et al.*, 2018 ▸; Vu & Ngo, 2018 ▸; Tandrup *et al.*, 2022 ▸). Increases in the θ_2_ angle and corresponding decreases in the θ_3_ angle are likely to also take place in the unbound state, although their statistical significance is obscured by the limits in precision. Changes in geometry are expected as the transition from Cu(II) to Cu(I) induces the loss of two exogenous ligands and accompanying changes in electronic structure; however, we clearly see in the room-temperature study that, on photoreduction at least, changes in the θ_2_ angle can be detected far before the loss of water ligands (Supplementary Fig. S4 and Supplementary Table S7). θ_2_ goes from 91.16° in the 1.5 kGy structure to 96.25° in the 88.5 kGy structures, while θ_3_ decreases from 175.25° to 166.95°.

The change in geometry of θ_2_ and θ_3_ in the unbound state, if real, may contribute to the higher affinity of the Cu(I) state for saccharides in LPMOs, which has been widely reported. The θ_2_ angle increases at 100 K from ∼94° to ∼98° and then again slightly (but not significantly) to ∼101° on the binding of saccharide to the Cu(I) state, while θ_3_ decreases slightly from 171° to 168° on reduction and continues to decrease to a statistically significantly different level of 161°. Thus, one can hypothesize that the reduction to Cu(I) brings the geometry closer to a high-affinity substrate-binding state, while also bringing the occupied orbitals of copper *d* character to the frontier, increasing the likelihood of electron transfer to incoming molecules, as shown by previous computational studies (with and without saccharide). Binding of saccharide, however, also induces an additional conformational change: loss of planarity at the copper site, as shown by a statistically significant increase in θ_T_ on going from either the LsAA9A_Cu(II)_LD (5.41°) or LsAA9A_Cu(I)_LD (4.90°) unbound state to LsAA9A_Cu(II)_Cell3_LD (12.4°). The effect of substrate binding in computational investigations, where the geometric changes in terms of loss of planarity were maintained, shows that substrate binding has a smaller effect on the electronic structure compared with copper reduction, but the energy difference between the highest occupied copper orbital and the remaining copper *d* orbitals is diminished. The same work suggested that in the presence of saccharide electron transfer to the H_2_O_2_ co-substrate may be facilitated (Wieduwilt *et al.*, 2024 ▸).

We confirm in this work that the Cu–Tyr O^η^ distance in LsAA9A_Cu(II)_Cell3_LD is about 0.2 Å shorter than in LsAA9A_Cu(II)_LD, with high statistical significance, but this work also suggests that this distance is shorter than in both the LsAA9A_Cu(I)_LD and LsAA9A_Cu(I)_Cell3_LD states, while the significance remains unclear. We suggest here that LsAA9A_Cu(II)_Cell3_LD may represent an ‘activated’ state trapped by the presence of Cl^−^; however, so far the role of the Tyr has mostly been associated with pathways of protection of LPMOs from oxidative damage, rather than a direct involvement in mechanism. The biological significance of the shortening of the Tyr–Cu distance in the LsAA9A mechanism thus remains to be clarified.

For LPMOs, few model systems have been found that allow crystallographic studies in complex with saccharide. Very recent advanced spectroscopic studies on a chitin-binding LPMO show a different approach to probe the subtle structural changes of these copper active sites (Joseph *et al.*, 2025 ▸), and confirm structural changes in a Cu(I) LPMO upon binding saccharide, which in turn influence the reactivity, as we have previously reported for LsAA9A (Wieduwilt *et al.*, 2024 ▸). Interestingly, however, some of the changes appear to be mediated by the acetyl group of the chitin substrate, which is not present in the cellulosic substrate of LsAA9A. This underscores that findings from LPMO model systems, while important, cannot necessarily be transferred to all LPMOs.

## Supplementary Material

PDB reference: LsAA9A, cryo temperature, low-dose structures of Cu(II) state, 9tcx

PDB reference: 9tcv

PDB reference: 9tcy

PDB reference: low-dose structures of Cu(II) state with cellotriose, 9tcz

PDB reference: 9td0

PDB reference: 9td1

PDB reference: low-dose structures of Cu(I) state, 9td6

PDB reference: 9td8

PDB reference: 9td9

PDB reference: low-dose structures of Cu(I) state with cellotriose, 9tdb

PDB reference: 9tdc

PDB reference: 9tdd

PDB reference: high-dose structures of Cu(I) state, 9tdh

PDB reference: 9tdi

PDB reference: high-dose structures of Cu(I) state with cellotriose, 9tdj

PDB reference: 9tdk

PDB reference: room temperature, multicrystal structure at 1.5 kGy, 9tde

PDB reference: at 88.5 kGy, 9tdf

Supplementary Figures and Tables. DOI: 10.1107/S2059798326005966/xh5063sup1.pdf

## Figures and Tables

**Table 1:** Data-collection and refinement statistics for the six LsAA9A single-crystal structures determined PDB entries 9tcx, 9tcv and 9tcy are LsAA9A_Cu(II)_LD structures. PDB entries 9tcz, 9td0 and 9td1 are LsAA9A_Cu(II)_Cell3_LD structures. Values in parentheses are for the highest resolution shell.

PDB code	9tcx	9tcv	9tcy	9tcz	9td0	9td1
Beamline	BioMAX, MAX IV, Lund	ID23-2, ESRF, Grenoble	BioMAX, MAX IV, Lund	BioMAX, MAX IV, Lund	BioMAX, MAX IV, Lund	BioMAX, MAX IV, Lund
Date of data collection	03/02/2024	04/04/2023	03/02/2024	03/02/2024	03/02/2024	03/02/2024
Autoprocessed dataset used	*autoPROC*	*grenades parallelproc*	*autoPROC* (manually cut the resolution in *XSCALE*)	*autoPROC* (manually cut the resolution in *XSCALE*)	*autoPROC* (manually cut the resolution in *XSCALE*)	*autoPROC* (manually cut the resolution in *XSCALE*)
Wavelength (Å)	0.9763	0.8731	0.9763	0.9763	0.9763	0.9763
Dose (Gy)	7.00 × 10^4^	5.37 × 10^4^	4.89 × 10^4^	6.40 × 10^4^	5.27 × 10^4^	4.24 × 10^4^
Space group	*P*4_1_	*P*4_1_	*P*4_1_	*P*4_1_	*P*4_1_	*P*4_1_
No. of molecules in asymmetric unit	1	1	1	1	1	1
*a*, *b*, *c* (Å)	48.9, 48.9, 109.6	49.5, 49.5, 109.6	48.5, 48.5, 109.5	48.1, 48.1, 108.9	48.3, 48.3, 109.6	48.0, 48.0, 108.8
α, β, γ (°)	90.0, 90.0, 90.0	90.0, 90.0, 90.0	90.0, 90.0, 90.0	90.0, 90.0, 90.0	90.0, 90.0, 90.0	90.0, 90.0, 90.0
Resolution (Å)	48.89–1.61 (1.79–1.61)	109.62–1.85 (1.89–1.85)	44.38–1.85 (1.90–1.85)	48.07–2.06 (2.11–2.06)	48.19–2.07 (2.12–2.07)	43.93–1.94 (1.99–1.94)
Completeness (%)	92.6 (71.5)	99.3 (90.6)	100 (99.7)	100.0 (99.8)	100.0 (100.0)	100.0 (99.9)
*R*_meas_ (%)	24.1 (112.5)	26.6 (192.0)	26.5 (88.6)	25.2 (98.3)	27.1 (130.7)	40.9 (187.8)
〈*I*/σ(*I*)〉	7.5 (1.3)	8.1 (1.1)	6.29 (1.02)	6.63 (1.0)	6.73 (1.50)	3.83 (0.26)
CC_1/2_ (%)	99.4 (74.4)	99.5 (74.8)	99.4 (62.7)	99.5 (73.9)	99.4 (57.1)	99.2 (62.6)
Observed reflections	281682 (6469)	304123 (16867)	265039 (11168)	195813 (8035)	164627 (12071)	256044 (19134)
Unique reflections	21896 (1095)	22367 (1271)	21593 (1585)	15277 (1116)	15289 (1120)	18227 (1366)
Multiplicity	12.9 (5.9)	13.6 (13.3)	12.3 (7.0)	12.8 (7.2)	10.8 (10.8)	14.0 (14.0)
DPI (Å)	0.20	0.22	0.24	0.30	0.29	0.29
*R*_work_ (%)	16.47	19.67	19.64	18.38	17.51	21.81
*R*_free_ (%)	21.4	22.88	23.71	23.22	21.8	26.53
R.m.s.d.						
Bond lengths (Å)	0.0092	0.0093	0.0087	0.0076	0.0103	0.0089
Bond angles (°)	1.5325	1.5163	1.5366	1.5577	1.7059	1.6602
Ramachandran statistics (%)						
Favored	94.0	94.8	94.4	94.4	94.0	94.4
Allowed	5.6	5.2	5.6	5.2	6.0	5.2
Outliers	0.4	0.0	0.0	0.4	0.0	0.4

**Table 2:** Data-collection and refinement statistics for the six LsAA9A single-crystal structures soaked with ascorbic acid determined PDB entries 9td6, 9td8 and 9td9 are LsAA9A_Cu(I)_LD structures. PDB entries 9tdb, 9tdc and 9tdd are LsAA9A_Cu(I)_Cell3_LD structures. Values in parentheses are for the highest resolution shell.

PDB code	9td6	9td8	9td9	9tdb	9tdc	9tdd
Beamline	ID30B, ESRF, Grenoble	ID30B, ESRF, Grenoble	ID30B, ESRF, Grenoble	ID23-2, ESRF, Grenoble	ID23-2, ESRF, Grenoble	ID23-2, ESRF, Grenoble
Date of data collection	07/06/2024	07/06/2024	07/06/2024	28/06/2024	28/06/2024	28/06/2024
Autoprocessed dataset used	*EDNA_proc*	*EDNA_proc*	*autoPROC*	*grenades parallelproc*	*grenades parallelproc*	*autoPROC*
Wavelength (Å)	0.8551	0.8551	0.8551	0.8731	0.8731	0.8731
Dose (Gy)	4.60 × 10^4^	4.60 × 10^4^	1.07 × 10^4^	1.80 × 10^4^	2.40 × 10^4^	2.5 × 10^4^
Space group	*P*4_1_	*P*4_1_	*P*4_1_	*P*4_1_	*P*4_1_	*P*4_1_
No. of molecules in asymmetric unit	1	1	1	1	1	1
*a*, *b*, *c* (Å)	48.7, 48.7, 109.1	48.7, 48.7, 109.3	48.6, 48.6, 109.2	48.2, 48.2, 109.2	48.3, 48.3, 109.4	48.3, 48.3, 109.4
α, β, γ (°)	90.0, 90.0, 90.0	90.0, 90.0, 90.0	90.0, 90.0, 90.0	90.0, 90.0, 90.0	90.0, 90.0, 90.0	90.0, 90.0, 90.0
Resolution (Å)	48.69–2.22 (2.30–2.22)	48.73–1.70 (1.76–1.70)	48.62–1.49 (1.52–1.49)	109.20–1.99 (2.04–1.99)	54.68–1.54 (1.57–1.54)	48.32–1.75 (1.78–1.75)
Completeness (%)	100.0 (99.9)	100.0 (100.0)	94.1 (84.1)	99.7 (97.1)	87.9 (36.3)	99.7 (95.9)
*R*_meas_ (%)	45.7 (351.6)	29.3 (798.1)	30.2 (680.4)	29.0 (194.7)	9.3 (82.1)	20.2 (100.4)
〈*I*/σ(*I*)〉	2.4 (0.3)	6.3 (0.2)	5.1 (0.2)	7.9 (1.3)	16.2 (1.5)	9.4 (2.7)
CC_1/2_ (%)	98.5 (62.8)	99.4 (55.3)	99.6 (50.0)	99.4 (57.1)	99.7 (73.1)	98.4 (35.1)
Observed reflections	145113 (10654)	375983 (30268)	526643 (22747)	237952 (16470)	347755 (2832)	204395 (5046)
Unique reflections	12572 (1220)	28034 (2758)	38557 (1722)	17110 (1167)	32424 (667)	25244 (1205)
Multiplicity	11.5 (8.7)	13.4 (11.0)	13.7 (13.2)	13.9 (14.1)	10.7 (4.2)	8.1 (4.2)
DPI (Å)	0.36	0.18	0.13	0.26	0.11	0.13
*R*_work_ (%)	21.21	18.54	17.42	18.17	12.40	13.26
*R*_free_ (%)	23.66	22.46	21.24	22.67	15.28	15.56
R.m.s.d.						
Bond lengths (Å)	0.0080	0.0098	0.0107	0.0079	0.0130	0.0126
Bond angles (°)	1.5380	1.5241	1.5962	1.6109	2.2045	2.0147
Ramachandran statistics (%)						
Favored	94.0	94.4	94.8	95.3	94.0	94.4
Allowed	5.6	5.5	5.2	4.7	6.0	5.6
Outliers	0.4	0.0	0.0	0.0	0.0	0.0

**Table 3:** One-way ANOVA results for selected geometric parameters at the copper active site for structures under four different experimental conditions *p*-values under 0.05 are marked in orange, while *p*-values under 0.001 are marked in red. The *F* in ANOVA is defined as the ratio of the mean-square variance between groups to the mean-square variance within groups.

Parameters	LsAA9A_Cu(II)_LD	LsAA9A_Cu(I)_LD	LsAA9A_Cu(II)_Cell3_LD	LsAA9A_Cu(I)_Cell3_LD	*F*	*p*-value
Distances						
Cu–His1 N^δ1^ (Å)	2.00 ± 0.0050	1.97 ± 0.0473	2.11 ± 0.0830	1.95 ± 0.0276	4.05	0.04010
Cu–His1 N^Am^ (Å)	2.17 ± 0.0342	2.24 ± 0.0404	2.22 ± 0.0770	2.30 ± 0.0656	3.10	0.07579
Cu–His78 N^ɛ2^ (Å)	2.04 ± 0.0443	2.00 ± 0.1044	2.07 ± 0.0627	1.96 ± 0.0173	1.98	0.18111
Cu–Tyr O^η^ (Å)	2.77 ± 0.0585	2.84 ± 0.0794	2.56 ± 0.0377	2.72 ± 0.0577	15.50	0.00043
Angles						
θ_1_ (°)	92.78 ± 1.8213	92.59 ± 1.7919	93.23 ± 1.3699	94.90 ± 0.7812	1.47	0.28028
θ_2_ (°)	94.22 ± 1.8658	97.92 ± 1.5974	90.75 ± 2.0279	100.56 ± 1.1092	20.96	0.00012
θ_3_ (°)	171.08 ± 4.2065	168.27 ± 0.6106	166.90 ± 2.1190	161.37 ± 1.8937	7.43	0.00662
θ_T_ (°)	5.41 ± 3.0266	4.90 ± 2.0561	12.40 ± 2.0702	10.22 ± 1.6256	9.03	0.00340

## Data Availability

The atomic coordinates and structure factors have been deposited in the Protein Data Bank (PDB) under accession codes 9tcx, 9tcv, 9tcy, 9tcz, 9td0, 9td1, 9td6, 9td8, 9td9, 9tdb, 9tdc, 9tdd, 9tdh, 9tdi, 9tdj, 9tdk, 9tde and 9tdf. All other data supporting the findings of this study are available within the article and its supporting information files.
