# Supplementary material for: Experimental estimation of copper-site geometry reproducibility in biologically relevant redox and saccharide-bound states of a model lytic polysaccharide monooxygenase
Source: Acta Crystallogr D Struct Biol. 2026 Jul 17;82(Pt 8):886–99. doi: 10.1107/S2059798326005966 (PMC13431639; doi:10.1107/S2059798326005966)
Supplement: Supplementary file 1 [file d-82-00886-sup1.pdf]

STRUCTURAL BIOLOGY

Volume 82 (2026)

Supporting information for article:

Experimental estimation of copper-site geometry reproducibility in biologically relevant redox and saccharide-bound states of a model LPMO

Zhiyu Huang, Qiuyi Wei, Jie Nan, Morten H. H. Nørholm, Zimeng Liu, Cristina Hernandez-Rollan, Katja S. Johansen and Leila Lo Leggio

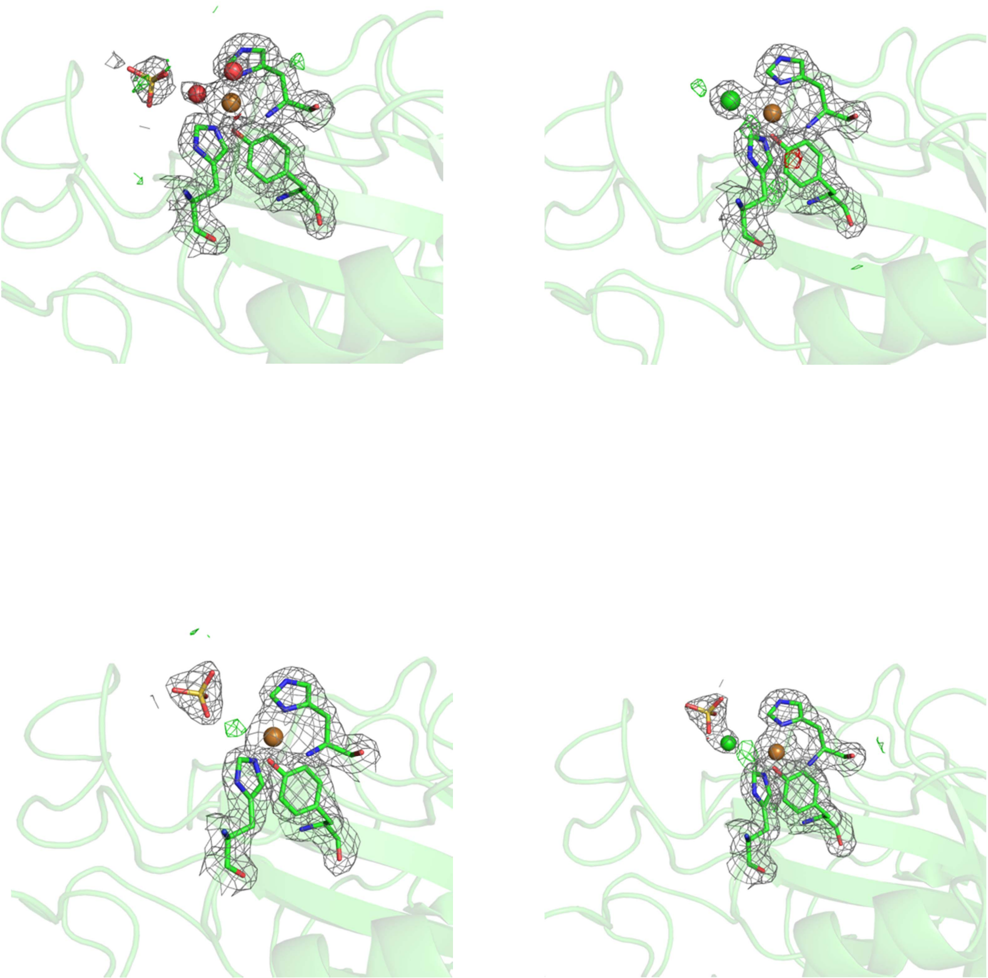

Figure S1 Active site of *Ls*AA9A_Cu(II)_LD_9TCV (left top), *Ls*AA9A_Cu(II)_Cell3_LD_9TD0 (right top), *Ls*AA9A_Cu(I)_LD_9TD6 and LsAA9A_Cu(I)_Cell3_LD_9TDB structures, showing the copper center and surrounding ligands. The 2Fo–Fc electron density map is contoured at 1.0 σ and displayed as a blue mesh. The Fo–Fc difference electron density map is contoured at ±3.0 σ, with positive density shown in red and negative density shown in green.

Table S1 Distances and angles of coordination atoms, equatorial and axial atoms with Cu active site in LsAA9A_Cu(II) and LsAA9A_Cu(II)_Cell3 low dose structures.

	PDB	Dose [Gy]	His1-$N_{\delta 1}$ [Å]	His1-N_{Am} [Å]	His78-$N_{\varepsilon 2}$ [Å]	O_{η}-Tyr [Å]	ϑ_1 [°]	ϑ_2 [°]	ϑ_3 [°]	ϑ_T [°]	Eq [Å]	Ax [Å]
LsAA9A_Cu(II)_LD	7pyl	1.49 x 10^4	2.00	2.13	2.02	2.68	91.80	94.90	172.60	3.30	1.94	2.63
LsAA9A_Cu(II)_LD	9TCX	7.00 x 10^4	2.01	2.21	2.10	2.80	90.72	92.05	176.32	2.41	2.02	2.64
LsAA9A_Cu(II)_LD	9TCV	5.37 x 10^4	2.00	2.15	2.02	2.80	94.07	96.41	167.23	7.26	1.87	2.74
LsAA9A_Cu(II)_LD	9TCY	4.89 x 10^4	2.00	2.17	2.00	2.79	94.53	93.51	168.17	8.67	2.11	2.73
Mean ± SD			2.00 ± 0.0050	2.17 ± 0.0342	2.04 ± 0.0443	2.77 ± 0.0585	92.78 ± 1.8213	94.22 ± 1.8658	171.08 ± 4.2065	5.41 ± 3.0266	1.99 ± 0.1034	2.69 ± 0.0580
LsAA9_Cu(II)_Cell3_LD	7pyu	1.49 x 10^4	1.96	2.11	1.99	2.55	91.30	91.70	169.20	10.40	2.33	
LsAA9_Cu(II)_Cell3_LD	9TCZ	6.40 x 10^4	2.09	2.29	2.06	2.61	94.46	87.92	164.87	14.96	2.54	
LsAA9_Cu(II)_Cell3_LD	9TD0	5.27 x 10^4	2.16	2.24	2.14	2.52	93.32	90.80	168.19	11.08	2.38	
LsAA9_Cu(II)_Cell3_LD	9TD1	4.24 x 10^4	2.08	2.24	2.09	2.55	93.84	92.60	165.35	13.16	2.44	
Mean ± SD			2.11 ± 0.0830	2.22 ± 0.0770	2.07 ± 0.0627	2.56 ± 0.0377	93.23 ± 1.3699	90.75 ± 2.0279	166.90 ± 2.1190	12.40 ± 2.0702	2.42 ± 0.0903	

Table S2 Distances and angles of coordination atoms with Cu active site in LsAA9A_Cu(I) and LsAA9A_Cu(I)_Cell3 low dose structures.

	PDB	Dose [Gy]	His1-$N_{\delta 1}$ [Å]	His1-N_{Am} [Å]	His78-$N_{\varepsilon 2}$ [Å]	O_{η}-Tyr [Å]	θ_1 [°]	θ_2 [°]	θ_3 [°]	θ_T [°]	Eq [Å]	Ax [Å]
LsAA9A_Cu(I)_LD	9TD6	4.60 x 10^4	1.95	2.26	1.95	2.87	94.64	96.2	168.36	4.20	4.02	3.51
LsAA9A_Cu(I)_LD	9TD8	4.60 x 10^4	1.93	2.26	1.93	2.75	91.82	98.22	167.62	7.21	2.91	3.44
LsAA9A_Cu(I)_LD	9TD9	1.07 x 10^4	2.02	2.19	2.12	2.9	91.32	99.36	168.83	3.27	3.92	3.33
Mean ± SD			1.97 ± 0.0473	2.24 ± 0.0404	2.00 ± 0.1044	2.84 ± 0.0794	92.59 ± 1.7919	97.92 ± 1.5974	168.27 ± 0.6106	4.90 ± 2.0561	3.62 ± 0.5014	3.43 ± 0.0900
LsAA9_Cu(I)_Cell3_LD	9TDB	1.80 x 10^4	1.92	2.36	1.96	2.66	95.71	101.47	159.2	11.54	3.22	
LsAA9_Cu(I)_Cell3_LD	9TDC	2.40 x 10^4	1.97	2.31	1.95	2.77	94.15	100.88	162.73	8.41	3.64	
LsAA9_Cu(I)_Cell3_LD	9TDD	2.5 x 10^4	1.95	2.23	1.96	2.74	94.84	99.32	162.17	10.73	3.65	
Mean ± SD			1.95 ± 0.0276	2.30 ± 0.0656	1.96 ± 0.0057	2.72 ± 0.0577	94.90 ± 0.7812	100.56 ± 1.1092	161.37 ± 1.8937	10.22 ± 1.6256	3.50 ± 0.2454	

Table S3 Statistical comparison of selected distances and angles around Cu in four groups of structure using Tukey-Kramer post hoc test and *t*-test (95% confidence interval). The yellow indicates significant difference according to Tukey-Kramer test. *p*-values under 0.05 are marked in orange, while *p*-values under 0.001 are marked in red according to *t*-test. Distances are to Cu atom. (Tukey-Kramer post hoc testing was not applicable for the Cu-Ax and Cu-Eq distances).

**Cu-His1-N$_{\delta 1}$ (Distance)** / **Cu-O$_{\eta}$-Tyr (Distance)**	LsAA9A_Cu(II)_LD	LsAA9_Cu(I)_LD	LsAA9_Cu(II)_Cell3_LD	LsAA9_Cu(I)_Cell3_LD
LsAA9A_Cu(II)_LD		0.18027	0.14329	0.01013
LsAA9_Cu(I)_LD	0.21945		0.01812	0.57995
LsAA9_Cu(II)_Cell3_LD	0.00094	0.00141		0.0055
LsAA9_Cu(I)_Cell3_LD	0.3529	0.10567	0.00581	
**θ_2 (Angle)** / **θ_3 (Angle)**	LsAA9A_Cu(II)_LD	LsAA9_Cu(I)_LD	LsAA9_Cu(II)_Cell3_LD	LsAA9_Cu(I)_Cell3_LD
LsAA9A_Cu(II)_LD		0.04014	0.04576	0.00356
LsAA9_Cu(I)_LD	0.31278		0.00401	0.07897
LsAA9_Cu(II)_Cell3_LD	0.12628	0.33683		0.00068
LsAA9_Cu(I)_Cell3_LD	0.01454	0.00386	0.01611	
**θ_T (Angle)**	LsAA9A_Cu(II)_LD	LsAA9_Cu(I)_LD	LsAA9_Cu(II)_Cell3_LD	LsAA9_Cu(I)_Cell3_LD
LsAA9A_Cu(II)_LD		0.81119	0.00885	0.05714
LsAA9_Cu(I)_LD			0.00507	0.02443
LsAA9_Cu(II)_Cell3_LD				0.19517
LsAA9_Cu(I)_Cell3_LD				
**Cu-Ax(Distance)** / **Cu-Eq(Distance)**	LsAA9A_Cu(II)_LD	LsAA9_Cu(I)_LD	LsAA9_Cu(II)_Cell3_LD	LsAA9_Cu(I)_Cell3_LD
LsAA9A_Cu(II)_LD		0.00004	-	-
LsAA9_Cu(I)_LD	0.00297		-	-
LsAA9_Cu(II)_Cell3_LD	0.0007	0.01071		-
LsAA9_Cu(I)_Cell3_LD	0.00009	0.78135	0.00041	
t-test p < 0.05				
t-test p < 0.001				

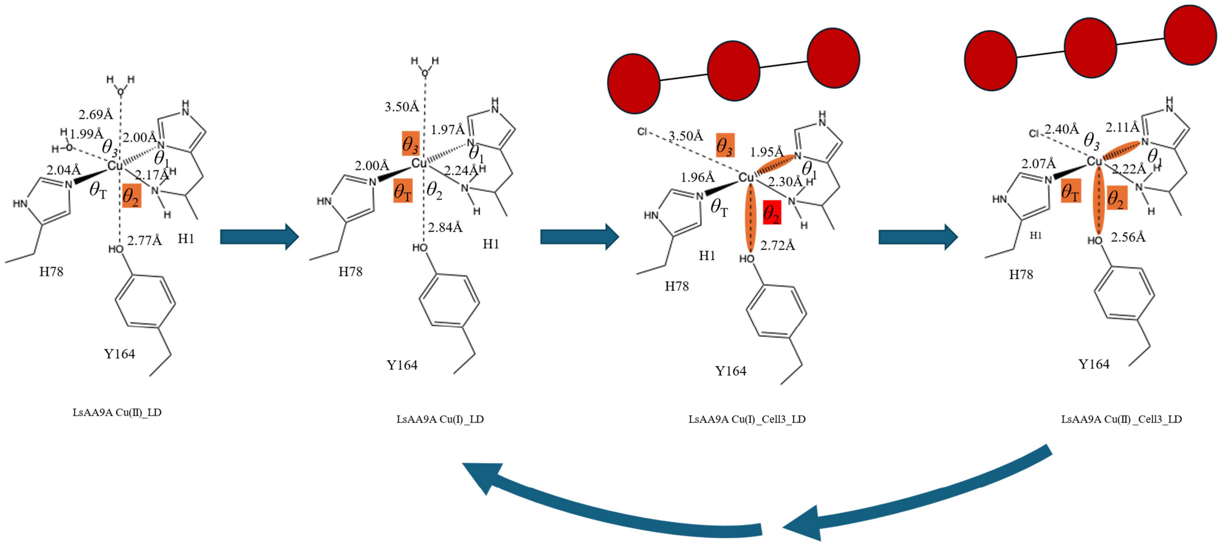

Figure S2 Schematic representation of the active site geometries of LsAA9A under four different conditions — Cu(II), Cu(I), Cu(I)-Cell3, and Cu(II)-Cell3, showing the mean distances of atoms coordinated to the Cu center. The figure illustrates selected significant structural parameters differences according to the *t*-test, highlighted in orange and red. Only differences between states that can be considered sequential according to the scheme in Figure 2A are highlighted: LsAA9A_Cu(II)_LD → LsAA9A_Cu(I)_LD → LsAA9A_Cu(I)_Cell3_LD → LsAA9A_Cu(II)_Cell3_LD (mimicking LsAA9A_Cu(II)_Cell3-HO—OH) →→ LsAA9A_Cu(I)_LD.

Table S4 Data Collection and Refinement Statistics for the 4 LsAA9A single crystal structures determined. 9TDH and 9TDI are LsAA9_Cu(I) high dose structures. 9TDJ and 9TDK are LsAA9_Cu(I)_Cell3 high dose structures. (*Highest resolution shell shown in parenthesis.)

	ID30B, ESRF, Grenoble; 07/06/2024- PDB: 9TDH	ID30B, ESRF, Grenoble; 07/06/2024- PDB: 9TDI	ID23-2, ESRF, Grenoble; 28/06/2024- PDB: 9TDJ	ID23-2, ESRF, Grenoble; 28/06/2024- PDB: 9TDK
Autoprocessed dataset used	EDNA_proc	EDNA_proc	grenades_parallelproc	EDNA_proc
Wavelength [Å]	0.8551	0.8551	0.8731	0.8731
Dose [Gy]	2.78×10^6	2.80×10^6	2.73×10^6	2.72×10^6
Space group	$P\,4_1$	$P\,4_1$	$P\,4_1$	$P\,4_1$
No. of mols/asymmetric unit	1	1	1	1
Cell parameters				
(a, b, c) [Å]	48.6, 48.6, 109.5	48.3, 48.3, 109.3	48.1, 48.1, 109.0	49.2, 49.2, 109.9
(α,β,γ) [°]	90.0, 90.0, 90.0	90.0, 90.0, 90.0	90.0, 90.0, 90.0	90.0, 90.0, 90.0
Resolution [Å]	48.63-1.34 (1.39-1.34)*	48.28-1.06 (1.10-1.06)*	54.53-1.86 (1.90-1.86)*	44.91-1.65 (1.71-1.65)*
Completeness [%]	100.0(100.0)	99.5 (95.3)	99.8 (99.6)	99.8 (99.7)
R_{meas} [%]	10.4 (255.8)	6.8 (146.3)	40.4 (580.0)	26.5 (140.3)
I/ σ (I)	14.5 (1.2)	17.5 (1.0)	6.3 (0.8)	3.7 (1.1)
$CC_{1/2}$ [%]	99.9 (58.8)	100.0 (52.7)	98.7 (47.1)	99.4 (63.8)
Observed reflections	778319 (76405)	1362755 (61299)	278703 (17175)	438995 (41890)
Unique reflections	56933 (5586)	112352 (10599)	20742 (1273)	31311 (3056)
Redundancy	13.7 (13.7)	12.1 (5.8)	13.4 (13.5)	14.0 (13.7)

DPI [Å]	0.08	0.04	0.26	0.14
R_{work} [%]	15.69	13.13	21.04	16.84
R_{free} [%]	18.65	15.21	25.89	19.09
RMSD				
Bond lengths [Å]	0.0131	0.0219	0.0082	0.0102
Bond Angles [°]	1.7381	2.0794	1.5677	1.6329
Ramachandran Statistics [#][%]				
Favored	94.4	95.3	94.0	95.7
Allowed	5.6	4.7	6.0	4.3
Outlier	0.0	0.0	0.0	0.0

Table S5 Distances and angles of coordination atoms with Cu active site in LsAA9_Cu(I) and LsAA9A_Cu(I)_Cell3 high dose structures.

	PDB	Dose [Gy]	His1-$N_{\delta 1}$ [Å]	His1-N_{Am} [Å]	His78-$N_{\varepsilon 2}$ [Å]	O_{η}-Tyr [Å]	θ_1 [°]	θ_2 [°]	θ_3 [°]	θ_T [°]
LsAA9_Cu(I)_HD	9TDH	2.78 x 10^6	1.87	2.28	1.88	2.82	94.41	98.6	166.04	4.99
LsAA9_Cu(I)_HD	9TDI	2.80 x 10^6	1.92	2.21	1.95	2.81	94.09	97.35	167.84	4.07
Mean ± SD			1.90 ± 0.0354	2.25 ± 0.0024	1.92 ± 0.0025	2.82 ± 0.0000	94.25 ± 0.2263	97.98 ± 0.8839	166.94 ± 1.2735	4.53 ± 0.6548
LsAA9A_Cu(I)_LD										
Mean ± SD			1.97 ± 0.0473	2.24 ± 0.0404	2.00 ± 0.1044	2.84 ± 0.0794	92.59 ± 1.7919	97.92 ± 1.5974	168.27 ± 0.6106	4.90 ± 2.0561
LsAA9A_Cu(I)_Cell3_HD	9TDJ	2.73 x 10^6	1.98	2.25	1.98	2.65	92.94	103.13	160.62	10.62
LsAA9A_Cu(I)_Cell3_HD	9TDK	2.72 x 10^6	1.94	2.35	1.94	2.71	95.28	100.01	162.32	8.78
Mean ± SD			1.96 ± 0.0269	2.30 ± 0.0742	1.96 ± 0.0297	2.68 ± 0.0431	94.11 ± 1.6525	101.57 ± 2.2069	161.47 ± 1.2014	9.70 ± 1.3011
LsAA9_Cu(I)_Cell3_LD										
Mean ± SD			1.95 ± 0.0276	2.30 ± 0.0656	1.96 ± 0.0057	2.72 ± 0.0577	94.90 ± 0.7812	100.56 ± 1.1092	161.37 ± 1.8937	10.22 ± 1.6256

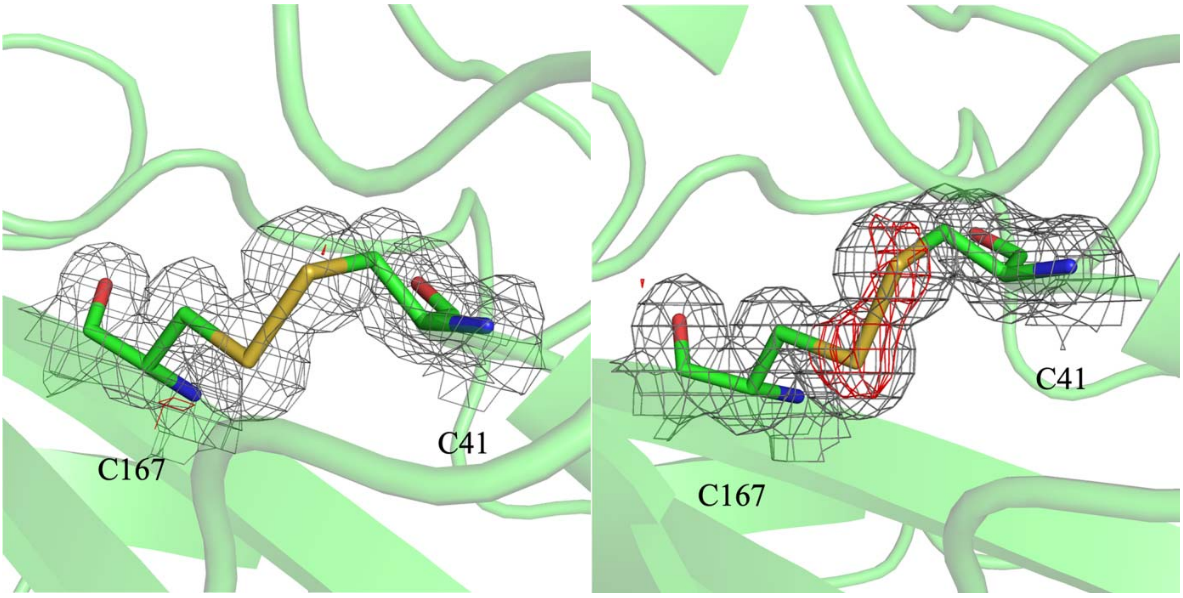

Figure S3 Disulfide bonds (Cys41-Cys167) in the LsAA9A_Cu(I)_LD_9TD9 (left) and LsAA9A_Cu(I)_HD_9TDH (right) structures, showing negative (red) difference density in the high dose dataset from 9TDH. $2F_o$-F_c electron density is shown at 1.0 σ contour level as blue mesh. F_o-F_c electron density is shown at ± 2.5σ contour level, with negative density shown as green mesh and positive density as red mesh.

Table S6 Data Collection and Refinement Statistics for the 2 LsAA9A room temperature structures determined. 9TDE is LsAA9A at 1.5 kGy and 9TDF is LsAA9A at 88.5 kGy. (*Highest resolution shell shown in parenthesis.)

	Micromax, MAXIV, Lund; 02/10/2024- PDB: 9TDE	Micromax, MAXIV, Lund; 02/10/2024- PDB: 9TDF
Wavelength [Å]	0.9544	0.9544
Space group	$P\,4_1$	$P\,4_1$
No. of mols/asymmetric unit	1	1
Cell parameters		
(a, b, c) [Å]	49.49, 49.48, 109.41	49.66, 49.66, 109.61
(α,β,γ) [°]	90.0, 90.0, 90.0	90.0, 90.0, 90.0
Resolution [Å]	50.00-1.75 (1.82-1.75)*	50.00-1.85 (1.98-1.85)*
Completeness [%]	95.8 (97.0)	93.6 (91.9)
R_{meas} [%]	26.0 (118.3)	28.5 (111.2)
I/ σ (I)	3.76 (0.97)	3.35 (0.98)
$CC_{1/2}$ [%]	97.2 (40.5)	96.3 (40.1)
Observed reflections	89347 (10014)	59294 (10964)
Unique reflections	25358 (2837)	21160 (3818)
Redundancy	3.52 (3.53)	2.80 (2.87)
DPI [Å]	0.17	0.21
R_{work} [%]	16.62	17.92
R_{free} [%]	19.16	21.22

RMSD		
Bond lengths [Å]	0.0097	0.0079
Bond Angles [°]	1.5184	1.4665
Ramachandran Statistics #[%]		
Favored	95.3	94.0
Allowed	43	6.0
Outlier	0.4	0.0

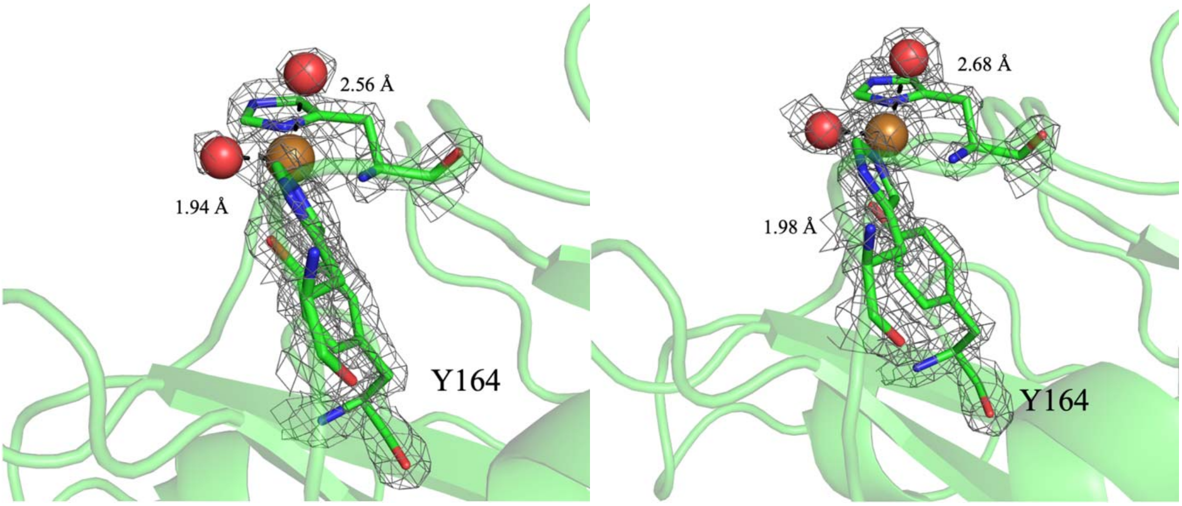

Figure S4 Electron density map of copper active from LsAA9A_RT_1.5kGy_9TDE (left) and LsAA9A_RT_88.5kGy_9TDF (right) by. In 9TDE, the equatorial and axial water molecules are coordinated to the copper at a distance of 1.94 Å and 2.56 Å respectively. In 9TDF, equatorial and axial water molecule are coordinated to the copper at a distance of 1.98 Å and 2.68 Å respectively. $2F_o$-F_c electron density is shown at 1.0 σ contour level as gray mesh. Brown sphere is copper, red sphere is H_2O. (*Sulphate and an additional water in 0.5 occupancy each are within hydrogen bonding of the equatorial water but are not shown here for simplicity in both structures).

Table S7 Distances and angles of coordination atoms with Cu active site in LsAA9A_RT_1_1-10_9TDE and LsAA9A_RT_1_291-300_9TDF room temperature structures by comparing with LsAA9A_Cu(II) and LsAA9A_Cu(I) structures at 100 K.

	PDB	Dose [Gy]	His1-$N_{\delta 1}$ [Å]	His1- N_{Am} [Å]	His78-$N_{\varepsilon 2}$ [Å]	O_{η}-Tyr [Å]	θ_1 [°]	θ_2 [°]	θ_3 [°]	θ_T [°]	Eq [Å]	Ax [Å]
LsAA9A_Cu(II)_LD												
Mean ± SD			2.00 ± 0.0050	2.17 ± 0.0342	2.04 ± 0.0443	2.77 ± 0.0585	92.78 ± 1.8213	94.22 ± 1.8658	171.08 ± 4.2065	5.41 ± 3.0266	1.99 ± 0.1034	2.69 ± 0.0580
LsAA9A_Cu(I)_LD												
Mean ± SD			1.97 ± 0.0473	2.24 ± 0.0404	2.00 ± 0.1044	2.84 ± 0.0794	92.59 ± 1.7919	97.92 ± 1.5974	168.27 ± 0.6106	4.90 ± 2.0561		3.43 ± 0.0900
LsAA9A_RT_1.5 kGy	9TDE	1.5 x10^3	1.98	2.17	2.02	2.68	91.84	91.16	175.25	3.69	1.94	2.56
LsAA9A_RT_88.5kGy	9TDF	4.5 x10^4	2.05	2.25	2.08	2.71	93.36	96.25	166.95	8.79	1.98	2.68

Table S8 Data Collection Statistics for the second RT photoreduction experiment - LsAA9A at average doses of 15 kGy (1.50 Å), 225kGy (1.90 Å) and 405kGy (2.60 Å).

	Micromax, MAXIV, Lund; 10/10/24-15kGy_1.50 Å	Micromax, MAXIV, Lund; 10/10/24-225kGy_1.90 Å	Micromax, MAXIV, Lund; 10/10/24-405kGy_2.60 Å
Wavelength [Å]	0.9544	0.9544	0.9544
Space group	$P\,4_1$	$P\,4_1$	$P\,4_1$
No. of mols/asymmetric unit	1	1	1
Cell parameters			
(a, b, c) [Å]	49.48, 49.48, 109.44	49.23, 49.23, 109.47	49.23, 49.23, 109.32
(α,β,γ) [°]	90.0, 90.0, 90.0	90.0, 90.0, 90.0	90.0, 90.0, 90.0
Resolution [Å]	100.00-1.50 (1.52-1.50)*	100.00-1.90 (1.98-1.90)*	100.00-2.60 (2.66-2.60)*
Completeness [%]	96.6 (90.4)	99.2 (99.6)	98.4 (98.4)
R_{meas} [%]	16.8 (91.2)	23.9 (87.7)	76.2 (302.7)
I/ σ (I)	4.83 (0.90)	5.33 (1.55)	4.98 (1.51)
$CC_{1/2}$ [%]	98.3 (43.2)	96.0 (51.7)	68.5 (31.3)
Observed reflections	223937 (5505)	114908 (13299)	43692 (2854)
Unique reflections	80279 (2915)	40189 (4653)	15529 (998)
Redundancy	2.79 (1.89)	2.86 (2.86)	2.81 (2.86)

Table S9 Data Collection and Refinement Statistics for the second RT photoreduction experiment - LsAA9A at average doses of 15 kGy, 225kGy, 405kGy and 585kGy. Note: all the highest resolution for these structures was set to 2.80 Å, the resolution obtained for the highest dose structure, in order to carry out meaningful comparisons of the density.

	Micromax, MAXIV, Lund; 10/10/24-15kGy_2.80 Å	Micromax, MAXIV, Lund; 10/10/24-225kGy_2.80 Å	Micromax, MAXIV, Lund; 10/10/24-405kGy_2.80 Å	Micromax, MAXIV, Lund; 10/10/24-585kGy_2.80 Å
Wavelength [Å]	0.9544	0.9544	0.9544	0.9544
Space group	$P\,4_1$	$P\,4_1$	$P\,4_1$	$P\,4_1$
No. of mols/asymmetric unit	1	1	1	1
Cell parameters				
(a, b, c) [Å]	49.48, 49.48, 109.44	49.23, 49.23, 109.47	49.23, 49.23, 109.32	49.41, 49.41, 109.70
(α,β,γ) [°]	90.0, 90.0, 90.0	90.0, 90.0, 90.0	90.0, 90.0, 90.0	90.0, 90.0, 90.0
Resolution [Å]	100.00-2.80 (2.85-2.80)*	100.00-2.80 (2.84-2.80)*	100.00-2.80 (2.90-2.80)*	100.00-2.80 (2.85-2.80)*
Completeness [%]	95.8 (97.3)	98.6 (99.2)	98.4 (98.8)	97.8 (98.8)
R_{meas} [%]	9.7 (12.5)	10.8 (18.3)	56.5 (206.6)	71.2 (275.2)
I/ σ (I)	10.81 (7.63)	9.63 (5.45)	5.86 (2.14)	3.10 (0.97)
$CC_{1/2}$ [%]	98.1 (97.4)	98.2 (94.6)	77.1 (41.3)	68.4 (30.2)
Observed reflections	35143 (1806)	34921 (1465)	34760 (3753)	35390 (1878)
Unique reflections	12229 (617)	12488 (512)	12450 (1291)	12492 (642)
Redundancy	2.87 (2.93)	2.80 (2.86)	2.79 (2.91)	2.83 (2.93)
DPI [Å]	0.42	0.42	0.50	0.49

R_{work} [%]	12.38	11.96	13.90	16.07
R_{free} [%]	16.63	16.85	19.87	22.22
RMSD				
Bond lengths [Å]	0.0064	0.0064	0.0066	0.0047
Bond Angles [°]	1.5060	1.4615	1.5691	1.3835
Ramachandran Statistics $^{\#}$[%]				
Favored	94.4	94.0	93.1	92.7
Allowed	4.7	5.6	6.4	6.9
Outlier	0.9	0.4	0.4	0.4

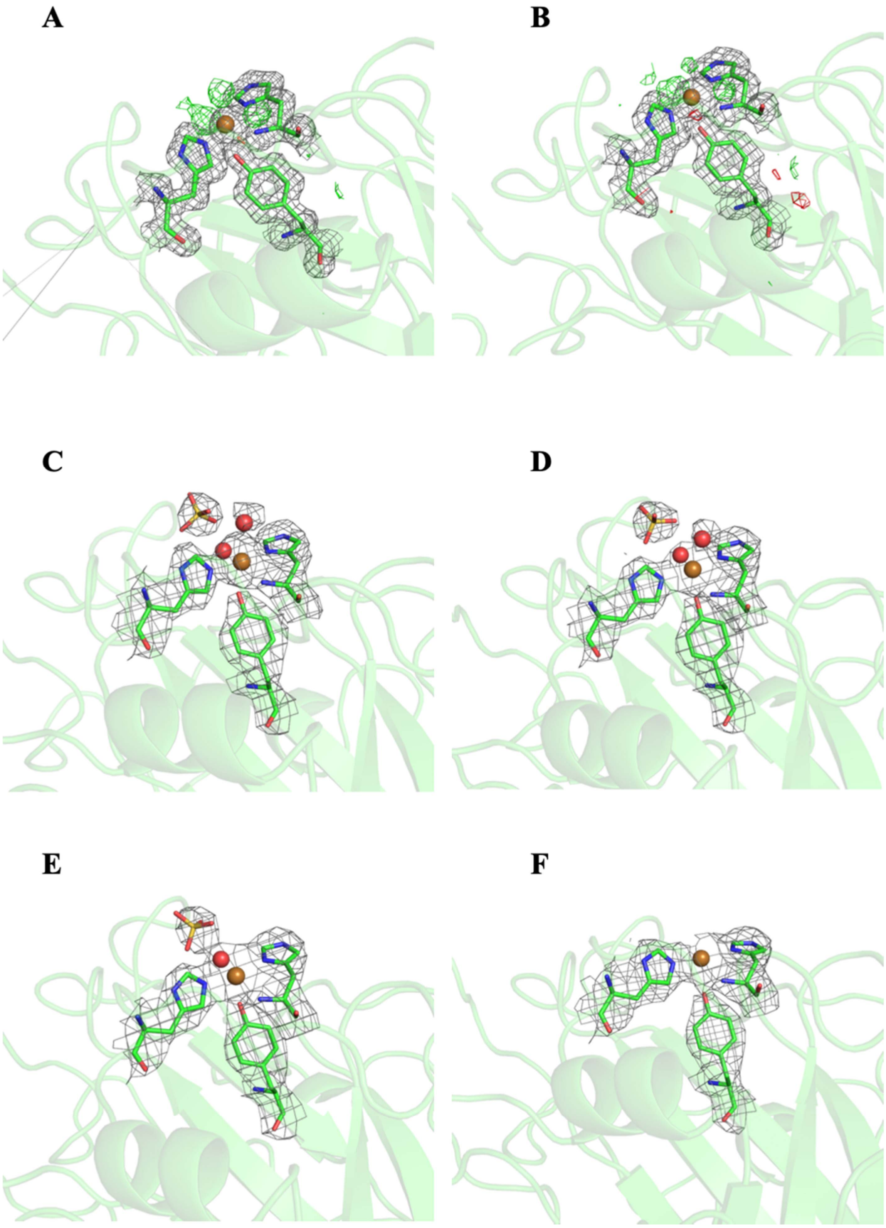

Figure S5 A is the active site of *Ls*AA9A_RT_15kGy at 1.50 Å and B is the active site of *Ls*AA9A_RT_225kGy at 1.90 Å, prior to extensive refinement. Difference density corresponding to close water ligands is clearly visible. Active site of *Ls*AA9A_RT_15kGy (C), *Ls*AA9A_RT_225kGy (D), *Ls*AA9A_RT_405kGy (E) and *Ls*AA9A_RT_585kGy (F) at 2.80 Å. Sulphate is shown in C, D and F at the equatorial position. The 2Fo–Fc electron density map is contoured at 1.0 σ and displayed as a blue mesh. The Fo–Fc difference electron density map is contoured at ± 3.0 σ, with positive density shown in red and negative density shown in green.
